# Building the Evidence Base of Blood-Based Biomarkers for Early Detection of Cancer: A Rapid Systematic Mapping Review

**DOI:** 10.1016/j.ebiom.2016.07.004

**Published:** 2016-07-06

**Authors:** Lesley Uttley, Becky L. Whiteman, Helen Buckley Woods, Susan Harnan, Sian Taylor Philips, Ian A. Cree, For the Early Cancer Detection Consortium

**Affiliations:** a The University of Sheffield, Regent Court, 30 Regent Street, Sheffield S1 4DA, UK; b Centre for Technology Enabled Health Research, Faculty of Health and Life Sciences, Coventry University, Coventry CV1 5FB, UK; c Warwick Medical School, University of Warwick, Coventry CV4 7AL, UK; d Department of Pathology, University Hospitals Coventry and Warwickshire, Coventry CV2 2DX, UK

**Keywords:** Cancer, Early detection, Biomarker, Assay, Diagnosis, Blood, Systematic review

## Abstract

**Background:**

The Early Cancer Detection Consortium is developing a blood-test to screen the general population for early identification of cancer, and has therefore conducted a systematic mapping review to identify blood-based biomarkers that could be used for early identification of cancer.

**Methods:**

A mapping review with a systematic approach was performed to identify biomarkers and establish their state of development. Comprehensive searches of electronic databases Medline, Embase, CINAHL, the Cochrane library and Biosis were conducted in May 2014 to obtain relevant literature on blood-based biomarkers for cancer detection in humans. Screening of retrieved titles and abstracts was performed using an iterative sifting process known as “data mining”. All blood based biomarkers, their relevant properties and characteristics, and their corresponding references were entered into an inclusive database for further scrutiny by the Consortium, and subsequent selection of biomarkers for rapid review. This systematic review is registered with PROSPERO (no. CRD42014010827).

**Findings:**

The searches retrieved 19,724 records after duplicate removal. The data mining approach retrieved 3990 records (i.e. 20% of the original 19,724), which were considered for inclusion. A list of 814 potential blood-based biomarkers was generated from included studies. Clinical experts scrutinised the list to identify miss-classified and duplicate markers, also volunteering the names of biomarkers that may have been missed: no new markers were identified as a result. This resulted in a final list of 788 biomarkers.

**Interpretation:**

This study is the first to systematically and comprehensively map blood biomarkers for early detection of cancer. Use of this rapid systematic mapping approach found a broad range of relevant biomarkers allowing an evidence-based approach to identification of promising biomarkers for development of a blood-based cancer screening test in the general population.

## Highlights

• There are a large number of biomarkers with potential utility for early cancer detection from blood samples
• Few biomarkers have been studied sufficiently with clinical validation to allow their use in combination for screening in the general population
• We used an iterative mapping review of 20,000 references, retrieving 3,990 relevant papers, and identified 788 markers in blood of potential use

Screening for cancer can save lives, but it is difficult to justify individual screening programmes for many cancer types. However, cancers of different types share many attributes, and markers of cancer biology found in the blood. We surveyed the literature to identify known biomarkers using a new mapping approach. With nearly 20,000 papers on the subject, we retrieved 3990 papers, and identified 788 markers in blood of potential use. Most have not been studied enough to justify their use in clinical practice. This evidence based approach should help us to develop a blood-based cancer screening test in the general population.

## 1 Introduction

Early detection of cancer results in improved survival (Etzioni et al., 2003, Wolf et al., 2010, McPhail et al., 2015). Cancers detected early require less extensive treatment and are less likely to have spread to other organs. Cancer diagnosis requires histological examination of tissue abnormalities detected by radiological, clinical or endoscopic examination of patients. Detection, as opposed to diagnosis, relies on screening a largely asymptomatic population to identify people who may be at higher risk of having cancer than others. Screening tests for cancer, or any other condition need to fulfil strict criteria to prevent the implementation of inappropriate screening, ensuring screening is cost effective and benefits patients. The criteria applied within the UK are listed at http://www.screening.nhs.uk/criteria, based on those developed by Wilson and Jungner (Cochrane and Holland, 1971, Wilson and Jungner, 1968). For early cancer detection, a blood-based screening test would have to be cost effective and demonstrate a meaningful clinical benefit which outweighs the harms associated with false positive, indeterminate results and overtreatment. This is clearly a major undertaking, and needs a multidisciplinary approach.

The Early Cancer Detection Consortium (ECDC) was established in 2012 in the United Kingdom and comprises 23 universities, their associated NHS hospitals, as well as other organisations and industry partners. The consortium was established to investigate whether a cost-effective screening test can be used in the general population to identify people with early cancers. Given the extensive literature on blood biomarkers for cancer, it is logical to explore the development of such a test using existing biomarkers that have the best evidence-base for cancer detection. A sensitive blood test for multiple tumour types could enable people with biomarker levels which are outside the typical range to receive further investigation and lead to earlier diagnosis of cancer at an asymptomatic stage when curative treatment is feasible. The next stage of the programme will involve analytical and clinical validation of these biomarkers in a case control study, from which a detection algorithm will be produced and validated for possible use as a generic cancer screen. Finally, a randomised controlled trial will be required to determine the clinical and cost-effectiveness of the resulting screening strategy.

Previous reviews in this area have understandably been limited in scope, usually restricted to one biomarker or well-defined group of potential markers, due to the enormous number of publications in the field. The aim of this study was therefore to establish the full range of candidate blood-based biomarkers with potential for the early detection of cancer, and map key characteristics of the tests.

## 2 Methods

To identify all relevant biomarkers, comprehensive searches and innovative methods to perform the mapping review were employed to cope with the sizeable body of relevant literature to be assessed within a short time-frame. The mapping review comprised the following stages: comprehensive literature searches; data mining techniques for rapid screening of the search records and; development of a customizable database of evidence to optimise the output from the mapping review. It was not considered sufficient simply to list evidence by reference or to name the biomarker once in a spreadsheet and continue searching until another new biomarker was found. Instead it was more useful and time-efficient to maintain the corresponding citations for each biomarker and record the basic characteristics of the study at the time of screening. This enabled a basic informative profile to be built for each biomarker identified in the mapping review.

This systematic review is registered with PROSPERO (no. CRD42014010827) and the methods have been structured around the PRISMA checklist (http://www.prisma-statement.org/).

### 2.1 Eligibility Criteria

Eligible studies included all English language studies from the past five years that investigated blood based biomarkers in more than 50 patients, see Table 1.

### 2.2 Search Strategy

To identify a comprehensive body of literature from which a list of candidate biomarkers could be generated, a broad search using keywords and subject headings was undertaken. The terms reflected the concepts of ‘diagnosis’, ‘markers’, ‘blood’ and ‘screening’ (see supplementary material). The keywords and subject headings were developed using a variety of collaborative methods between Information Specialists and Systematic Reviewers at the University of Sheffield and researchers at the University of Warwick.

A scoping search was performed and assessed for appropriateness. Additionally, key journal articles and abstracts in Medline were retrieved and assessed to obtain relevant subject headings and keywords. Clinical input was sought from members of the ECDC to verify and validate the chosen keywords. For the full search, relevant free-text, keyword and thesaurus terms were combined using Boolean operators and translated into database specific syntax. Full searches were limited to English language, humans and publication dated from 2010 to May 2014. The databases searched were Medline and Medline in Process, Embase, CINAHL, Cochrane Library (including Cochrane Database of Systematic Reviews, DARE, CENTRAL, HTA, NHS EED), Science Citation Index Expanded, Conference Proceedings Citation Index - Science, Book Citation Index – Science, and Biosis Previews.

The initial search strategy was broad and inclusive. As a result, a large number of relevant records were obtained. Preliminary validation by consulting experts in the field indicated that the search was sensitive and no missing relevant literature was identified.

### 2.3 Sifting and Data Mining

The results of the initial searches were imported into a Reference Manager database. To identify an exhaustive list of biomarkers, retrieved records were searched iteratively within the Reference Manager database, using keywords to select potentially relevant titles. Titles and abstracts of this selection of citations were scrutinised for names and descriptions of biomarkers that met (or potentially met) the selection criteria (see Table 1). The citations were tagged to indicate that they had been viewed, to enable their exclusion from further searches. Relevant citations were exported to a Microsoft Access database which was customised to allow data extraction of relevant key information for each biomarker that was available from the corresponding study abstracts.

The data mining process within the main database included the following restrictions (see Box 1):

To ensure a comprehensive capture of all relevant biomarkers, a further validation stage was performed. Relevant reviews identified during the search were used to check for additional biomarkers not generated by the data mining process. ECDC members were invited to recommend papers that they believed to be relevant to the mapping review.

### 2.4 Data Collection

Each biomarker occupied a record with a unique identifier number in a customised Microsoft Access database which stored the number of associated papers, the abstract and reference details; associated synonyms and acronyms; types of cancers and study design; keywords used to retrieve the abstract during data mining; assays used to measure the biomarker, where reported; category to which the biomarker was assigned (e.g. auto-antibodies); and the sample types used, where reported (e.g. serum, plasma or whole blood).

### 2.5 Results

**Fig. 1:**
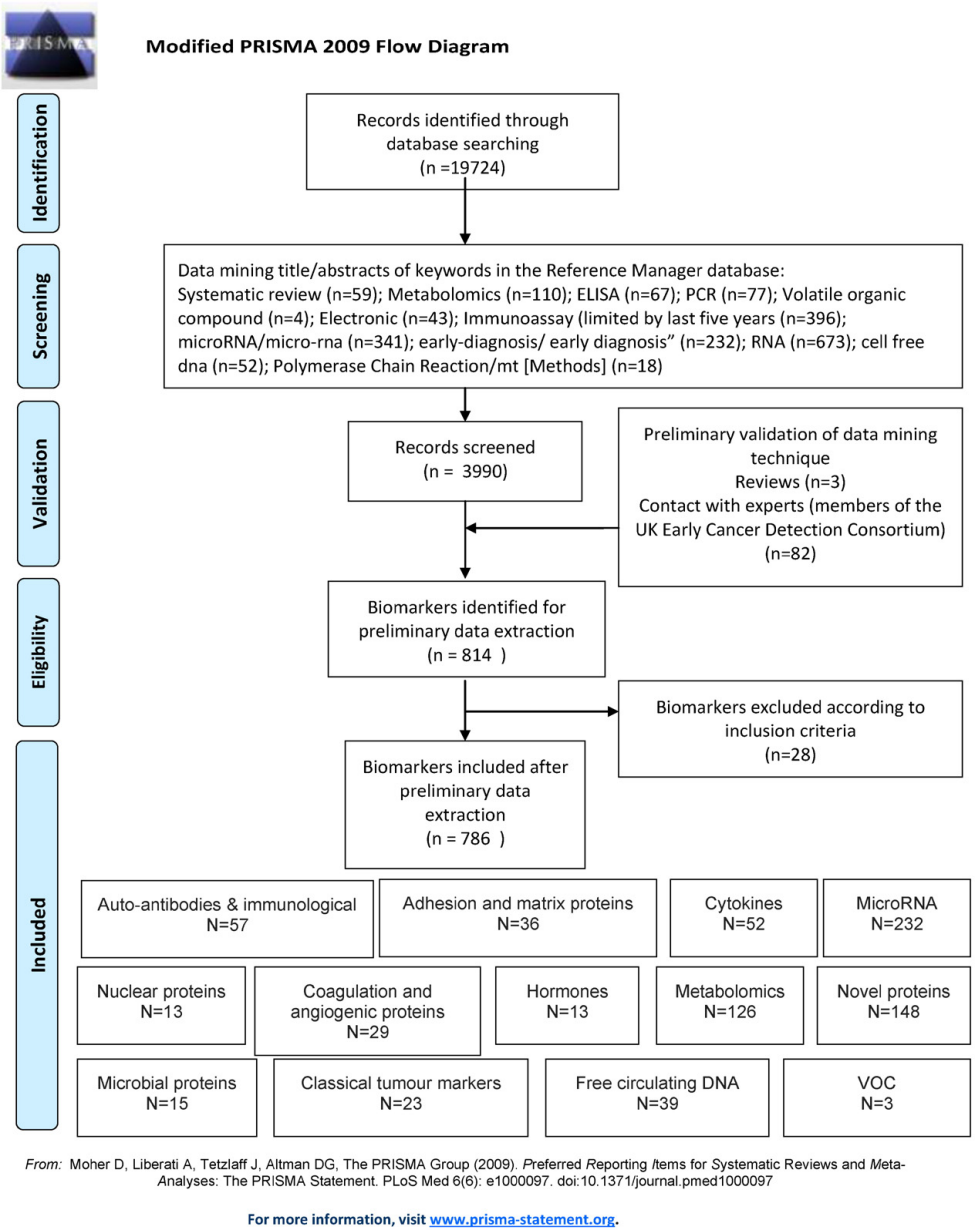
Modified PRISMA 2009 flow diagram.

After duplicates were removed, 19,724 records were yielded from the comprehensive searches. Using data mining, 3990 titles and abstracts were retrieved from the 19,724 records for full scrutiny. Data mining is the process of pulling a subset of records from a large, unwieldy dataset. The subset of 3990 abstracts was reviewed in order to generate a list of biomarkers which are potentially relevant to early identification of cancer using blood. A full breakdown of the keywords used and the number of corresponding records retrieved can be seen in Fig. 1. During the validation process, three relevant reviews were consulted for the identification of any additional biomarkers. No further biomarkers were identified either from these reviews or from the consultation of ECDC members.

A total of 814 biomarkers were identified as potentially relevant to the review question and were subjected to further scrutiny, identifying duplicates and miss-classified biomarkers during a process of data cleaning and categorising the biomarkers into groups or families. These groups are currently arranged by molecular function in order to map the biomarkers by biological origin. Further research using this methodology and database into the empirical application and validation of each biomarker will allow the biomarkers to be grouped by clinical utility such as cancer type or platform. However, we have performed this analysis for colorectal cancer (Table 2) and lung cancer (Table 3) to illustrate how these data could be used to define cancer-specific biomarkers. This resulted in a final total of 788 biomarkers, grouped into 13 initial categories (see Supplementary Table 1, Supplementary Table 2, Supplementary Table 3, Supplementary Table 4, Supplementary Table 5, Supplementary Table 6, Supplementary Table 7, Supplementary Table 8, Supplementary Table 9, Supplementary Table 10, Supplementary Table 11, Supplementary Table 12, Supplementary Table 13) as follows:

1. Adhesion and matrix proteins (*n* = 36). The expression of molecules involved in adhesion or in formation of the connective tissue matrix around cancer cells differ from non-neoplastic cells and appear in blood. Early work included collagen breakdown products, which are produced as a result of increased collagen turnover, but are not specific to particular tumour types (Paterson et al., 1991, Berruti et al., 1995). Collagens are metabolised by matrix metalloproteinase proteins (MMPs), these in turn are antagonised by tissue inhibitors of matrix metalloproteinases (TIMPs) (Roy et al., 2009). Both MMPs and TIMPs are represented in this group. Turnover of other matrix proteins is altered in cancer: vimentin (Ludwig et al., 2009), laminin (Schechter & Lopes, 1990) and tenascin are included in the list. Cancer cells have increased motility compared with non-neoplastic cells, and show altered expression of adhesion molecules. EpCAM, e-cadherin, and e-selectin are represented as blood biomarkers in the list (Beije et al., 2015, Hauselmann and Borsig, 2014, Gires and Stoecklein, 2014). Following review, a total of 18 were removed, including one duplicate entry.
2. Auto-antibodies and immunological markers (*n* = 59). The majority of entries in this category relate to auto-antibodies. These have been described for a wide variety of proteins within cancer, notably nuclear proteins such as P53 and other nuclear proteins, and occur in many cancers (Middleton et al., 2014). Immunological markers of interest include CRP, usually regarded as a marker of inflammation.
3. Classical Tumour Markers. A total of 23 markers were included in the ‘classical’ tumour marker group. This includes those used widely in practice, including CEA, CA125, CA15-3, CA19-9, AFP, and PSA. Markers of lesser utility, such as LDH and HE4 were also included. It should be noted that several of these (CA15-3 and CA19-9) refer to different epitopes of the same antigen, MUC1, which also came up in our searches.
4. Coagulation & angiogenic proteins. Of the 29 proteins in this category, the majority had relatively little evidence for their utility in early cancer detection. The markers can be sub-categorised into those connected to angiogenesis (e.g. VEGF, PlGF, Angiopoietins) and coagulation (e.g. plasminogen activating proteins and kallikreins). Annexins were included in this group, though they are more often thought of as apoptosis associated proteins.
5. Cytokines, chemokines and insulin-like growth factors. 52 biomarkers were included in this group. They include a wide range of cytokines and soluble receptors. Evidence for these is limited, but they represent an interesting group of proteins abnormal in cancer, measurement of which is likely to reflect the profound local immune suppression and systemic alteration of immunity present in cancers.
6. Circulating-free DNA. This is usually abbreviated as cfDNA, though increasingly the term circulating tumour DNA (ctDNA) is used. While DNA is clearly a single biomarker, 39 individual biomarkers representing genes or alterations of most interest were identified in this group, though in essence any mutation of gene methylation marker identified would be part of this group. While the first descriptions of cfDNA used PCR (Lo, 2001a, Lo, 2001b), many recent papers apply multi-analyte methods, including next generation sequencing (Coco et al., 2015, Rothe et al., 2014, Couraud et al., 2014), to the study of cfDNA to detect mutations of potential diagnostic significance. Though as yet few have used this for early detection.
7. Hormones. While 13 biomarkers were assigned to this category, only Corticosteroid-binding globulin survives more stringent searches (Wu et al., 2012). Hormone levels are not thought to be reliable markers of cancer.
8. Metabolomics. A large number of metabolites are known to be altered in cancer, as the result of changes in energy, lipid, amino acid, and protein metabolism. We identified 126 individual markers, many of which were measured in concert by mass spectroscopy within several studies (Cross et al., 2014, Hasim et al., 2013).
9. MicroRNA and other RNAs. There are now over 1000 human miRNA species known, a large number of these have been studied in cancer. While the majority have been looked at in tissue, there is considerable interest in their possible use as a liquid biopsy, our list of 232 biomarkers in this group reflects this. They are rarely measured alone: most use some form of array strategies for measurement, most studies concentrate on single cancer types (Fortunato et al., 2014, Clancy et al., 2014).
10. Novel Proteins. A large number of protein biomarkers, often identified by mass spectroscopy or 2D gel electrophoresis, were hard to categorise. These were grouped as novel proteins and represent a diverse group of 148 biomarkers. Examples include alpha-2-heremans-schmid-glycoprotein (AHSG) (Dowling et al., 2012) and galectin (Gromov et al., 2010) in breast cancer.
11. Nuclear proteins. A group of 13 nuclear protein biomarkers were assigned to this category, though some markers within the novel protein group are of nuclear origin. Circulating nucleosomes are included in this group as they are usually detected by ELISA (Holdenrieder et al., 2014).
12. Microbial proteins (*n* = 15). A small number of Epstein-Barr Virus (EBV) and Human Papilloma Virus (HPV) proteins and their antibodies have been studied as early cancer biomarkers in blood, based on the detection of EBV DNA in cancer patients (Lo, 2001b). Helicobacter antibodies also fall into this group.
13. Volatile Organic Compounds (VOC). Only three biomarkers, all small metabolites, were assigned to this category, which it could be argued forms part of the metabolite group. It is however measured differently.

## 3 Discussion

We systematically searched the literature from the last five years to identify potential blood biomarkers for cancer (Hanahan and Weinberg, 2011, Cree, 2011). The data mining process retrieved 3990 citations from the initial 19, 724 records, screening the abstracts of these citations identified 814 biomarkers that may be relevant. After data-cleaning, 788 biomarkers were fitted into 13 categories as described above as having potential for use as early cancer detection biomarkers present within blood samples. Biomarkers were grouped by molecular function. Further analysis such as grouping by cancer type may be possible only once the utility of each biomarker has been reviewed independently. As this is a mapping review, it is not possible to speculate the definitive clinical utility for each biomarker. Most studies reviewed tended to concentrate on single common cancers, and few papers show evidence of a systematic approach to biomarker discovery but were limited by the clinical samples and techniques of their laboratories.

The conduct of large systematic reviews is challenging, yet not all biomedical questions can be reduced to the size where standard methodologies for systematic review are thought reasonable. We have therefore taken a data mining approach to map blood biomarkers that may be suitable for the early detection of cancer using the search tools available within the reference management software. As with any approach to reviewing literature that falls short of a full systematic review, there is a balance between rigour and expenditure of time and resources. In this case, the aim was not to identify all relevant literature (as would be the case in a systematic review of efficacy), but rather all relevant biomarkers. It should be noted that the database does not hold the full text of the articles referenced and is restricted to titles, abstracts and keywords. Full text searching using machine learning algorithms could eventually provide a better solution.

In this instance, to allow a thorough search of the large dataset of biomarker literature and ensure an efficient approach to managing the data, we used data mining tools available within the reference management software. This allowed us to retrieve potentially relevant records, extract data relating to relevant biomarkers, and validate the process through adjunctive searches of reviews and through contact with an extensive network of experts. While the use of experts to validate the data may be regarded as subjective, it was a necessary step in validation of the searches and the multidisciplinary consortium involved in this work covers a large range of expertise. The limitation to studies published after 2009 could have skewed the data towards new technologies, and therefore reviews were included to mitigate the risk of ignoring older methodologies. Despite this limitation, it is notable that proteomic biomarkers, a more mature technology, formed a large proportion of the biomarkers found. Furthermore, it is possible that many of those biomarkers that have received less attention more recently did so because they were found to have limited utility in subsequent studies. We used conservative selection criteria that may have resulted in the inclusion of irrelevant biomarkers, but will have minimised the chance of relevant biomarkers being excluded. As such, we are confident that our methodology is fit for purpose and will have had high sensitivity for the identification of relevant biomarkers.

Limiting the mapping review to abstracts may have excluded studies identifying multiple potential biomarkers if such biomarkers were only mentioned in the main text. This is unlikely to occur in the field of emerging and promising biomarkers where the aim is to highlight the biomarker and technology to the audience. However, the vagueness of the abstracts of many papers is a challenge, as is the generally poor quality of study design. Even some larger scale studies from major groups do not include controls and few studies were powered to examine multiple biomarkers in comparison with existing tumour markers. The majority of cases (when described) are from patients with advanced disease, and this is a major concern for those interested in early detection: there is no guarantee that biomarkers identified in patients with advanced disease are relevant to those with early disease. There is certainly a need to improve the quality of papers on early detection using tools such as those available from the EQUATOR network (http://www.equator-network.org).

Our intention is to use the list of biomarkers identified by this review to generate a set of biomarkers that can be subjected to analytical validation within pathology blood science laboratories, then clinically validated within a large, prospective, multicentre clinical study to develop a generic cancer testing strategy for subsequent clinical trial. The primary aim is to produce a screening test strategy for cancer that does more good than harm at reasonable cost. Good includes decreased morbidity and mortality from early detection, diagnosis and treatment of cancers, while harm is usually regarded as significant risk of overdiagnosis, and consequent overtreatment. The entire strategy needs to be cost effective to achieve eventual approval from the UK National Screening Committee (NSC), which defines 22 criteria according to the condition, the test, the treatment and the screening programme (http://www.screening.nhs.uk/criteria) based on those developed by Wilson & Jungner (1968).

Within the list, there are some interesting results. Firstly, it is clear that current tumour markers, which considered in isolation, few would regard as sensible diagnostic tests in patients with a possible diagnosis of cancer, are collectively quite good at detection if used concurrently. The bulk of the work on this comes from one group in Barcelona (Molina et al., 2012), with other important contributions from others (Barak et al., 2010). The validation of biomarkers needs a point of reference, for direct comparison and it is clear that tumour marker lists used by Molina et al. (2012) represent such a standard. We would encourage those active in the field to use this list as their comparator for future work to allow comparison between studies.

The biomarkers can be grouped by the technology used for their detection. Taken to its logical conclusion, this results in a reduction of the thirteen groups above to seven groups as outlined in Box 3.

The ability of protein measurement to be multiplexed by immunoassay arrays or mass spectroscopy means that all proteins, including auto-antibodies, can be measured simultaneously. Simple panels with few analyses tend to be less expensive and have greater potential for high throughput. DNA and RNA can be detected rapidly and inexpensively by polymerase chain reaction (PCR) technologies, and there is evidence from multiple studies that the level of cfDNA has potential as a generic cancer marker. However, PCR is limited in the number of targets that can be detected at one time, and by the small amount of material present in patients with small tumours, which does not permit large numbers of tests to be performed without recourse to sequencing or large panels. Sequencing has the potential to detect large numbers of mutations, adding specificity, and could have utility in reflex testing. It is currently an expensive option, but costs of sequencing are decreasing rapidly, while technologies available are improving their capability at almost the same pace.

Metabolomics is of considerable interest, with a large literature to support it. While larger molecules require mass spectroscopy to measure their presence, smaller molecules can be detected in gas phase in the head space of blood samples using inexpensive sensor technologies. We believe that this relatively new option may have considerable potential to act as a generic test. There are a number of other tests that do not fit immediately into one of these seven categories: nucleosome assays are one such example, and are being used as potential screening tests.

The concept of combining high sensitivity/low specificity tests with reflex low sensitivity/high specificity tests to detect cancers early (Cree, 2011), seems feasible from the results we have obtained. We need to combine biomarkers with high sensitivity for screening the general population with biomarkers of high specificity to determine the relevance of the screening results. The next task is clearly to try this in practice to determine its real potential for early cancer detection, and to determine the best analytical methods to process the data for individual patients. Our preferred strategy is to examine the biomarkers in each category in greater detail, and undertake direct comparison of these biomarkers in a large cohort of samples following independent analytical validation. In our view, the same caveats around retrospective studies apply to biomarker validation as they do to drug trials: the potential for bias from sample collections is high and large prospective studies are necessary. This review is therefore the first step in an ambitious programme of work which will inevitably require careful evaluation of clinical, cost and ethical implications at each stage. However, there is no doubt that if such an approach to early cancer detection proved successful, it could be invaluable.

## 4 Conclusion

This ground-breaking study is the first to systematically and comprehensively map blood biomarkers for early detection of cancer and will inform an innovative research project to identify, validate and implement new generic blood screening tests for early cancer detection in the general population.

The following are the supplementary data related to this article.

Supplementary Table 1 Adhesion and matrix proteins. Supplementary Table 1

Supplementary Table 2 Auto-antibodies & immunological markers. Supplementary Table 2

Supplementary Table 3 Classical tumour markers. Supplementary Table 3

Supplementary Table 4 Coagulation and angiogenesis molecules. Supplementary Table 4

Supplementary Table 5 Cytokines, chemokines and insulin-like growth factors. Supplementary Table 5

Supplementary Table 6 Circulating-free DNA. Supplementary Table 6

Supplementary Table 7 Hormones. Supplementary Table 7

Supplementary Table 8 Metabolic markers. Supplementary Table 8

Supplementary Table 9 MicroRNA and other RNAs. Supplementary Table 9

Supplementary Table 10 Novel proteins. Supplementary Table 10

Supplementary Table 11 Nuclear proteins. Supplementary Table 11

Supplementary Table 12 Microbial proteins. Supplementary Table 12

Supplementary Table 13 Volatile organic compounds. Supplementary Table 13

## Authors Contributions

IC, SH, BW, and STP designed the study. Searches were performed HBW. LU performed the mapping review with input from the ECDC. The draft manuscript was prepared by LU, IC and BW. All authors agreed the final version.

## Declaration of Competing Interests

The authors LU, IC, SH, STP, BW, HBW have no conflicts of interest to declare. The ECDC has grant funding for early cancer biomarker research from Cancer Research UK and involves the following companies GE Healthcare, Life Technologies, Abcodia, Nalia, and Perkin-Elmer. Individual ECDC members have declared their interests to the ECDC secretariat.

## Funding

This work was conducted on behalf of the Early Cancer Detection Consortium, within the programme of work for work packages 1 & 2. The Early Cancer Detection Consortium is funded by Cancer Research UK under grant number: C50028/A18554.

## Figures and Tables

**Table 1:** Eligibility criteria for the systematic mapping review.

Inclusion criteria	Exclusion criteria
English language studies	Studies published in non-English language
Studies within the last five years (2010–2014)	Studies from 2009 or older
Controlled studies	No healthy control group
Validation studies	Derivative studies from included papers
Cancer detection/diagnosis	Prognosis or prediction (treatment response) associated markers
50 or more patients	Less than 50 patients
Biomarkers measured in blood	Tissue or other bodily fluid samples
	Abstracts of panels which do not state which biomarkers are studied
	Citation titles without abstracts

**Table 2:** Colorectal cancer specific biomarkers from all 13 categories.

Biomarker categories	ID no	Biomarker	Acronym	Cancer
Adhesion and matrix proteins	7	Clusterin	CLI	Colorectal
12	Ep cell adhesion module (GA733-2)	EpCAM (GA733-2)	Colorectal
22	Metallopeptidase inhibitor 1	TIMP1; TIMP-1	Colorectal
Auto-antibodies & immunological markers	2	Anti-p53 antibodies	p53; serum p53 antibodies; p53-Abs; p-53-AAB; Anti-p53Ab	Colorectal
19	Anti-heat shock protein 60	HSP60	Colorectal
40	IL2RB	IL2RB	Colorectal
Classical tumour markers	3	Carcinoembryonic antigen	CEA	Colorectal
8	Carbohydrate antigen 19-9	CA19-9; CA199	Colorectal
Coagulation and angiogenesis molecules	2	Vascular endothelial growth factor	VEGF	Colorectal
8	Kininogen-1	Kininogen-1	Colorectal
23	Endothelial cell-specific molecule-1	ESM-1	Colorectal
27	Thrombomodulin	THBD-M	Colorectal
28	Annexin A3	ANXA3	Colorectal
Cytokines, chemokines and insulin-like growth factors	3	Interleukin 8	IL-8	Colorectal
17	Insulin-like growth factor-binding protein-2	IGFBP-2	Colorectal
26	Brain-derived neurotrophic factor	BDNF	Colorectal
28	Interleukin-1ra	IL-1ra	Colorectal
50	TNFAIP6	TNFAIP6	Colorectal
Circulating-free DNA	3	Adenomatous polyposis coli	APC	Colorectal
9	Septin 9	Septin 9	Colorectal
17	Methylation of CYCD2	CYCD2	Colorectal
18	Methylation of HIC1	HIC1	Colorectal
19	Methylation of PAX 1	PAX 1	Colorectal
20	Methylation of RB1	RB1	Colorectal
21	Methylation of SRBC	SRBC	Colorectal
34	Line1 79 bp	Line1 79 bp	Colorectal
35	Line1 300 bp	Line1 300 bp	Colorectal
36	Alu 115 bp	Alu 115 bp	Colorectal
37	Alu 247 bp	Alu 247 bp	Colorectal
Hormones		Nil	Nil	
Metabolic markers	1	Plasma glucose levels	Plasma glucose levels	Colorectal
5	3-Hydroxypropionic acid and pyruvic acid	3-Hydroxypropionic acid and pyruvic acid	Colorectal
6	Alanine	l-Alanine, glucuronoic lactone	Colorectal
7	l-Glutamine	Glutamine	Colorectal
8	Sarcosine	Sarcosine	Colorectal
11	Choline	Phosphatidylcholine; (PC) (34 : 1)	Colorectal
12	Phosphatidylinositol	Phosphatidylinositol	Colorectal
17	l-Valine	Valine	Colorectal
18	l-Threonine	Threonine	Colorectal
19	1-Deoxyglucose	1-Deoxyglucose	Colorectal
20	Glycine	Glycine	Colorectal
21	MACF1	MACF1	Colorectal
22	Apolipoprotein H	APOH; beta-2-glycoprotein	Colorectal
23	Alpha-2-macroglobulin	A2M	Colorectal
24	Immunoglobulin lambda locus	IGL@	Colorectal
25	Vitamin D-binding protein	VDB	Colorectal
30	2-Hydroxyglutarate	2-Hydroxyglutarate	Colorectal
34	2-Hydroxybutyrate	2-Hydroxybutyrate	Colorectal
35	Aspartic acid	Aspartic acid	Colorectal
36	Kynurenine	Kynurenine	Colorectal
37	Cystamine	Cystamine	Colorectal
50	Tricarboxylic acid	TCA	Colorectal
53	2-Aminoethanesulfonic acid	Taurine	Colorectal
54	Lactate	Lactate	Colorectal
55	Phosphocholine	Phosphocholine	Colorectal
56	Proline	Proline	Colorectal
57	Phenylalanine	Phenylalanine	Colorectal
102	Oleamide	Oleamide	Colorectal
111	Leukocyte methylated cytosine 5	5-mC	Colorectal
116	Plasma choline-containing phospholipids	Plasma phospholipids	Colorectal
120	Palmitic amide	Palmitic amide	Colorectal
121	Hexadecanedioic acid	Hexadecanedioic acid	Colorectal
122	Octadecanoic acid	Octadecanoic acid	Colorectal
123	Eicosatrienoic acid	Eicosatrienoic acid	Colorectal
124	Lysophosphatidylcholine 18:2	LPC(18:2)	Colorectal
125	Lysophosphatidylcholine 16:0	LPC(16:0)	Colorectal
MicroRNA and other RNAs	5	let-7g		Colorectal
15	miR-126	miR-126	Colorectal
32	miR-135b	miR-135b	Colorectal
36	miR-141	miR-141	Colorectal
38	miR-143	miR-143	Colorectal
39	miR-145	miR-145	Colorectal
57	miR-17-3p	miR-17-3p	Colorectal
68	miR-18a	miR-18a	Colorectal
71	miR-191-5p	miR-191-5p	Colorectal
94	miR-20a	miR-20a	Colorectal
95	miR-21	miR-21	Colorectal
125	miR-29a	miR-29a	Colorectal
187	miR-548as-3p	miR-548as-3p	Colorectal
195	miR-601	miR-601	Colorectal
210	mir-760	mir-760	Colorectal
214	miR-885-5p	miR-885-5p	Colorectal
219	miR-92a	miR-92a	Colorectal
231	U6 snRNA (U6)	U6 snRNA (U6)	Colorectal
Novel proteins	15	Microtubule-associated protein RP/EB family member 1	MAPRE1	Colorectal
16	Leucine-rich alpha-2-glycoprotein	LRG1	Colorectal
56	Alpha-enolase	Alpha-enolase	Colorectal
62	Betaine	Betaine	Colorectal
72	CACNAG1	CACNAG1	Colorectal
82	Colon cancer specific antigen-2	CCSA-2	Colorectal
88	C9orf50-M	C9orf50-M	Colorectal
89	CLEC4D	CLEC4D	Colorectal
90	LMNB1	LMNB1	Colorectal
91	PRRG4	PRRG4	Colorectal
92	VNN1	VNN1	Colorectal
103	Dermokine-beta	DK-beta	Colorectal
105	Seprase	Seprase	Colorectal
126	Serum amyloid A	SAA	Colorectal
132	Lipocalin 2	Lipocalin 2	Colorectal
Nuclear proteins	2	k-ras	k-ras	Colorectal
Microbial proteins		Nil	Nil	
Volatile organic compounds	1	Phenyl methylcarbamate	Phenyl methylcarbamate	Colorectal
2	Ethylhexanol	Ethylhexanol	Colorectal
3	6-*t*-Butyl-2,2,9,9-tetramethyl-3,5- decadien-7-yne	6-*t*-Butyl-2,2,9,9-tetramethyl-3,5-decadien-7-yne	Colorectal

**Table 3:** Example for lung cancer and mesothelioma specific biomarkers from all 13 categories.

Biomarker categories	ID no	Biomarker	Acronym	Cancer
Adhesion and matrix proteins	2	Calreticulin	CRT	Lung
7	Clusterin	CLI	Lung
8	Cross-linked telopeptide of type I collage	ICTP	Lung
9	E-cadherin	E-cadherin; soluble E-cadherin (sE-cad)	Lung
10	E-cadherin gene CDH1	CDH1	Lung
11	E-selectin	E-selectin; sE-selectin	Lung
19	Matrix metalloproteinase-2	MMP2	Lung
29	Soluble L-selectin	sL-selectin	Lung
31	Surfactant protein-D	SP-D	Lung
Auto-antibodies & immunological markers	2	Anti-p53 antibodies	p53; serum p53 antibodies; p53-Abs; p-53-AAB; Anti-p53Ab	Lung
3	Anti-survivin antibodies	Survivin/anti-survivin antibodies	Lung
6	Inosine monophosphate dehydrogenase	IMPDH	Lung
8	Immunoglobulin G	IgG	Lung
12	Anti-livin	Livin/anti-livin antibodies	Lung
22	C-reactive protein	CRP	Lung
28	Anti-Krebs von Lungren-6	KL-6	Lung
30	Anti-ubiquillin	Ubiquillin	Lung
32	Alpha-crystallin IgG antibodies	Alpha-crystallin antibodies	Lung
37	CD30	CD30	Lung
38	CD63	CD63	Lung
43	NY-ESO-1	NY-ESO-1	Lung
44	CAGE	CAGE	Lung
45	GBU4-5	GBU4-5	Lung
46	SOX2	SOX2	Lung
47	HuD	HuD	Lung
48	IgM autoantibodies	IgM autoantibodies	Lung
55	Anti-hydroxysteroid-(17-alpha)-dehydrogenase		Lung
56	Anti-triosephosphate isomerase		Lung
Classical tumour markers	2	Cancer antigen 15-3	CA15-3; CA 15-3	Lung
3	Carcinoembryonic antigen	CEA	Lung
6	Human epididymis protein 4	HE4	Lung
9	Squamous cell carcinoma antigen	SCCA; SCC-ag	Lung
11	Cytokeratin fragment 19	CYFRA 21-1	Lung
12	Neuron Specific Enolase	NSE	Lung
14	Progastrin-releasing peptide	proGRP	Lung
22	HER2	HER2; AB_HER2; 36 HER2 negative; erbb-2; soluble human epidermal growth factor receptor 2 (sHER2)	Lung
Coagulation and angiogenesis molecules	1	Urokinase plasminogen activator	uPA/uPAR/suPAR	Lung
2	Vascular endothelial growth factor	VEGF	Lung
10	Endothelin-1	ET-1	Lung
13	Angiopoietin-2	Angiopoietin-2; Apo-2	Lung
14	Thrombospondin-1	THBS1	Lung
15	Plasminogen activator inhibitor	Plasminogen activator inhibitor	Lung
19	Endostatin	Endostatin	Lung
21	Annexin A1	ANXA1 mNRA	Lung
24	C4d	C4d	Lung
25	Annexin A2	ANXA2	Lung
Cytokines, chemokines and insulin-like growth factors	7	Tumour necrosis factor [alpha]	TNF[alpha]; DcR3	Lung
10	Macrophage migration inhibitory factor	MIF	Lung
18	Hepatocyte growth factor	HGF	Lung
19	Insulin-like growth factor binding protein	IGFBP-3	Lung
20	Granulocyte-colony stimulating factor	G-CSF	Lung
21	Interleukin 3	IL-3	Lung
22	Stem cell factor	SCF	Lung
25	C-C motif chemokine 5	C-C motif chemokine 5	Lung
28	Interleukin-1ra	IL-1ra	Lung
29	Monocyte chemotactic protein-1	MCP-1	Lung
31	Midkine	MK; MDK	Lung
38	IRF1	IRF1	Lung
51	Macrophage inflammatory protein 4	MIP-4	Lung
52	Megakaryocyte potentiating factor	MPF	Mesothelioma
Circulating-free DNA	1	Microsatellite alterations at FHIT	FHIT	Lung
2	Microsatellite alterations at loci on chromosome 3	3p loci	Lung
3	Adenomatous polyposis coli	APC	Lung
4	CHD1	CHD1	Lung
5	*O*(6)-Methyl-guanine-DNA methyltransferase	MGMT	Lung
6	DCC	DCC	Lung
7	RASSF1A	RASSF1A	Lung
8	absent in melanoma 1	AIM1; beta/gamma crystallin domain-containing protein 1	Lung
Hormones	9	Progesterone receptor B	PRB	Lung
13	Prolactin	Prolactin	Lung
Metabolic markers	6	Alanine	l-Alanine, glucuronoic lactone	Lung
26	Leucine	Leucine; isoleucine	Lung
27	Histidine	Histidine	Lung
28	Tryptophan	Tryptophan	Lung
29	Ornithine	Ornithine	Lung
38	Lactic acid	Lactic acid	Lung
39	Glycelic acid	Glycelic acid	Lung
40	Glycolic acid	Glycolic acid	Lung
87	NG1A2F	NG1A2F	Lung
89	N-glycopeptides	Glycopeptides	Mesothelioma
102	Oleamide	Oleamide	Lung
103	Long chain acyl carnitines	Long chain acyl carnitines	Lung
104	Lysophosphatidylcholine 18:1	LPC(18:1)	Lung
105	Lysophosphatidylcholine 20:4	LPC(20:4)	Lung
106	Lysophosphatidylcholine 20:3	LPC(20:3)	Lung
107	Lysophosphatidylcholine 22:6	LPC(22:6)	Lung
108	Serum metabolite 16:0/1	SM(16:0/1)	Lung
115	Ferritin	FTL	Lung
MicroRNA and other RNAs	7	miR-103	miR-103	Mesothelioma
14	miR-1254	miR-1254	Lung
15	miR-126	miR-126	Mesothelioma
20	miR-128b	miR-128b	Lung
29	miR-133a	miR-133a	Lung
35	miR-140	miR-140	Lung
38	miR-143	miR-143	Lung
41	miR-1468	miR-1468	Lung
43	miR-146b-3p	miR-146b-3p	Lung
50	miR-155	miR-155	Lung
53	miR-15b	miR-15b	Lung
60	miR-181c	miR-181c	Lung
61	miR-182	miR-182	Lung
68	miR-18a	miR-18a	Lung
80	miR-197	miR-197	Lung
95	miR-21	miR-21	Lung
98	miR-212	miR212	Lung
106	miR-220	miR-220	Lung
108	miR-221	miR-221	Lung
111	miR-23a	miR-23a	Lung
122	miR-27b	miR-27b	Lung
135	miR-30c-1*	miR-30c-1*	Lung
145	miR-330	miR-330	Lung
147	miR-331	miR-331	Lung
152	miR-339-5p	miR-339-5p	Lung
157	miR-345	miR-345	Lung
158	miR-346	miR-346	Lung
172	miR-377	miR-377	Lung
180	miR-484	miR-484	Lung
188	miR-548b	miR-548b	Lung
189	miR-550	miR-550	Lung
190	miR-566	miR-566	Lung
192	miR-574–5p	miR-574–5p	Lung
197	miR-616*	miR-616*	Lung
198	miR-625*	miR-625*	Mesothelioma
203	miR-656	miR-656	Lung
204	miR-660	miR-660	Lung
213	miR-876-3p	miR-876-3p	Lung
218	miR-92	miR-92	Lung
221	miR-939	miR-939	Lung
224	miR-let-7	let-7	Lung
Novel proteins	3	Haptoglobin	HP	Lung
21	CD9	CD9	Lung
22	CD81	CD81	Lung
39	HMGA1	HMGA1	Lung
40	TFDP1	TFDP1	Lung
41	SUV39H1	SUV39H1	Lung
42	RBL1	RBL1	Lung
43	HNRPD	HNRPD	Lung
58	Anterior gradient 2	AGR2	Lung
63	Pentraxin-3	PTX3	Lung
67	Lysyl oxidase	LOX	Lung
75	Death receptor 3	DR3	Lung
76	Membrane-spanning 4 domain subfamily A from the multigene family of proteins involved in signal transduction of which CD20 is one member	MS4A	Lung
93	Heat shock protein 90 alpha	HSP90alpha	Lung
94	Leucine-rich repeats and immunoglobulin-like domains 3	LRIG3	Lung
95	Pleiotrophin	Pleiotrophin	Lung
96	Protein kinase C iota type	PRKCI	Lung
97	Repulsive Guidance Molecule C	RGM-C	Lung
98	Stem Cell Factor soluble Receptor	SCF-sR	Lung
99	YES	YES	Lung
116	HMGB1	HMGB1	Mesothelioma
119	Carbohydrate antigen 50	CA50	Lung
125	Cytokeratin fragment 21.1	Cytokeratin fragment 21.1	Lung
126	Serum amyloid A	SAA	Lung
128	Carbohydrate antigen 211	CA211	Lung
146	Endoplasmic reticulum protein-29	ERP29	Lung
Nuclear proteins	3	Isocitrate dehydrogenase 1	IDH1	Lung
4	p53 messenger RNA	p53 mRNA	Lung
10	E2F6	E2F6	Lung
13	Variant Ciz1	Ciz1	Lung
Microbial proteins	6	Epstein-Barr virus-induced gene 3	EBI3	Lung
Volatile organic compounds		Nil	Nil	

## Box 1 Data mining process restrictions.

**Table:**

Restriction	Justification
1. *Searches limited to last five years.*	To ensure that the biomarkers identified and their associated evidence is current and relevant, searches were restricted to records published in the last five years (from 2010 to May 2014).
2. *Data mining technique employed*, *as opposed to screening all references*	Data mining involved interrogation of search results using relevant keywords (Box 2) to search within the database of total records for batches of references. Keywords were identified through consultation with ECDC members for known technologies, and for other potentially relevant terms. Keywords for similar concepts (e.g. synonyms for a specific biomarker) were grouped and searched together. Keywords expected to retrieve citations of high relevance were prioritised over those with less obvious relevance. Further keywords were identified by the review team by consideration of indexing keywords and content of studies identified as relevant.
3. *One reviewer performed the data mining*.	One reviewer screened the references to generate the list of biomarkers using the data mining technique. A single reviewer screening approach was mitigated for by the examination review papers and consultation with ECDC membership during a later validation phase. An inclusive approach to inclusion was adopted to minimise inappropriate exclusions.
4. *Pragmatic inclusion criteria*	Titles without abstracts were not included. Equally abstracts of primary studies or reviews which did not name a biomarker were not included. Titles and abstracts retrieved from each batch of references associated with each keyword were assessed against the eligibility criteria in Table 1.

Alt-text: Box 1

## Box 2 Keywords used in data mining process.

Keywords used: “systematic review”; “metabolomics”; “ELISA”; “PCR”; “volatile organic compound”; “electronic”; “immunoassay”; “microRNA”; “early diagnosis”; RNA”; “biomarkers”; and “fluorescence”.

Alt-text: Box 2

## Box 3 Seven biomarker groupings based on technology used for detection

Seven biomarker groupings 1. Existing tumour marker panels (current standard for comparison); 2. Auto-antibodies. 3. Circulating free DNA from the tumour (ctDNA); 4. Circulating MicroRNA (miRNA); 5. Volatile Organic Compounds (VOC); 6. Mass Spectroscopy (MS); 7. Other biomarkers.

Alt-text: Box 3
