# Supplementary material for: Building the Evidence Base of Blood-Based Biomarkers for Early Detection of Cancer: A Rapid Systematic Mapping Review
Source: eBioMedicine. 2016 Jul 6;10:164–73. doi: 10.1016/j.ebiom.2016.07.004 (PMC5006664; doi:10.1016/j.ebiom.2016.07.004)
Supplement: Supplementary Table 1 — Adhesion and matrix proteins. [file mmc1.docx]

**Supplementary Table 1: Adhesion and Matrix Proteins**

| **No** | **Biomarker** | **Acronym** | **Cancer** |
| --- | --- | --- | --- |
| 1 | asporin | asporin | Prostate |
| 2 | Calreticulin | CRT | Lung |
| 3 | carcinoembryonic cell adhesion molecule 6 | CEAM6 | Bilary tract |
| 4 | cartilage oligomeric matrix protein | cartilage oligomeric matrix protein | Prostate |
| 5 | Cell adhesion module 17.1 | CAM17.1 | Pancreatic |
| 6 | cellular fibronectin | cellular fibronectin | Ovarian |
| 7 | clusterin | CLI | Colorectal, Hepatocellular, Lung |
| 8 | cross-linked telopeptide of type I collage | ICTP | Lung |
| 9 | E-cadherin | E-cadherin; soluble E-cadherin (sE-cad) | Breast, Lung, Prostate |
| 10 | E-cadherin gene CDH1 | CDH1 | Gastric, Lung |
| 11 | E-selectin | E-selectin; sE-selectin | Lung |
| 12 | Ep Cell Adhesion Module (GA733-2) | EpCAM (GA733-2) | Colorectal |
| 13 | extracellular matrix protein-1 | extracellular matrix protein-1 | Ovarian |
| 14 | gelsolin | gelsolin | Bone |
| 15 | hyaluronic acid | HYAL-1; HYuA | Bladder, Gastrointestinal, Mesothelioma |
| 16 | lamin B1 | LMNB1 | Hepatocellular |
| 17 | laminin | laminin | Gastrointestinal |
| 18 | Matrix metalloproteinase 9 | MMP9 | Prostate |
| 19 | matrix metalloproteinase-2 | MMP2 | Lung |
| 20 | matrix metalloproteinase-7 | MMP7 | Bilary tract, Ovarian |
| 21 | mesothelin | mesothelin | Mesothelioma, Ovarian, Pancreatic |
| 22 | metallopeptidase inhibitor 1 | TIMP1; TIMP-1 | Colorectal, Pancreatic |
| 23 | Osteopontin | OPN | General, Hepatocellular, Mesothelioma, Ovarian, Pancreatic |
| 24 | PAM4 | PAM4 | Pancreatic |
| 25 | periostin | periostin | Endometrial, Hepatocellular |
| 26 | procollagen type I N-propeptide | P1NP | Bone, Breast |
| 27 | serum tissue inhibitor of metalloproteinases 4 | TIMP-4 | Ovarian |
| 28 | soluble intercellular adhesion molecule-1 | sICAM-1 | Bladder |
| 29 | soluble L-selectin | sL-selectin | Lung |
| 30 | soluble mesothelin-related protein/peptide | SMRP | General, Mesothelioma, Ovarian |
| 31 | surfactant protein-D | SP-D | Lung |
| 32 | Tartrate-resistant acid phosphatase 5b | TRACP5b | Bone |
| 33 | tenascin C | TNC | Ovarian, Pancreatic |
| 34 | type IV collagen (amino-terminal propeptide of procollagen type IV) | type IV collagen (amino-terminal propeptide of procollagen type IV) | Gastric |
| 35 | vimentin | VIM | Hepatocellular |
| 36 | Fibulin-3 | Fib3 | Mesothelioma |
