# Supplementary material for: Building the Evidence Base of Blood-Based Biomarkers for Early Detection of Cancer: A Rapid Systematic Mapping Review
Source: eBioMedicine. 2016 Jul 6;10:164–73. doi: 10.1016/j.ebiom.2016.07.004 (PMC5006664; doi:10.1016/j.ebiom.2016.07.004)
Supplement: Supplementary Table 2 — Auto-antibodies & immunological markers. [file mmc2.docx]

**Supplementary Table 2: Auto-antibodies & Immunological Markers**

| **No** | **Biomarker** | **Acronym** | **Cancer** |
| --- | --- | --- | --- |
| 1 | Anti-EpCAM antibody | Ber-Ep4 | Mesothelioma |
| 2 | Anti-p53 antibodies | p53; serum p53 antibodies; p53-Abs; p-53-AAB; Anti-p53Ab | Breast, Colorectal, Lung, Oesophageal |
| 3 | anti-survivin antibodies | survivin/ anti-survivin antibodies | General, Lung, Prostate |
| 4 | Anti-Mucin 1 antibodies | MUC1 | Ovarian |
| 5 | Anti-Mucin 5AC | MUC5AC | Bilary tract |
| 6 | Inosine monophosphate dehydrogenase | IMPDH | Lung |
| 7 | Anti-Heat shock protein 70 | HSP70 | Leukemia, Prostate |
| 8 | Immunoglobulin G | IgG | General, Lung |
| 9 | immunoglobulin A | IgA | General |
| 10 | Anti-extracellular protein kinase | ECPKA | General |
| 11 | H. pylori antibodies (antilysate and antiCagA) | H. pylori CagA+; CagA | Gastric |
| 12 | Anti-livin | livin/ anti-livin antibodies | Lung |
| 13 | B7-H4 | B7-H4 | Ovarian |
| 14 | immunoglobulin M | IgM | Ovarian |
| 15 | Anti-Thyroglobulin | Tg; TgAb | Thyroid |
| 16 | anti-tumor-associated antigens | anti-TAA | Hepatocellular |
| 17 | anti-TIF1gamma antibody | anti-TIF1gamma antibody | General |
| 18 | C-terminal-truncated form of C3a | C-terminal-truncated form of C3a | Breast |
| 19 | Anti-Heat Shock Protein 60 | HSP60 | Colorectal |
| 20 | anti-peroxiredoxin | PRDX1; PDRX2 | Ovarian, Prostate |
| 21 | Anti-glial fibrillary acidic protein | GFAP | Glioma |
| 22 | C-reactive protein | CRP | Cervical, General, Lung, Ovarian |
| 23 | Anti-apolipoprotein A1 | apolipoprotein A1; ApoA-1 | Endometrial, Gastric, Ovarian |
| 24 | CD61 | CD61 | Prostate |
| 25 | B7H3 | B7H3 | Prostate |
| 26 | anti-Ku86 | anti-Ku86 | Hepatocellular |
| 27 | Sialyl Lewis X | sLeX | Breast |
| 28 | Anti-Krebs von Lungren-6 | KL-6 | Lung |
| 29 | Anti-mitogen-activated protein kinase 9 | MAPK9 | Pancreatic |
| 30 | Anti-ubiquillin | ubiquillin | Lung |
| 31 | astrocyte elevated gene-1 auto-antibodies | AEG-1-Abs | General |
| 32 | Alpha-crystallin IgG antibodies | Alpha-crystallin antibodies | Lung |
| 33 | soluble MHC class I chain-related molecules A | sMICA | Pancreatic |
| 34 | soluble MHC class I chain-related molecules B | sMICB | Pancreatic |
| 35 | CD23 | CD23 | Lymphoma |
| 36 | CD27 | CD27 | Lymphoma |
| 37 | CD30 | CD30 | Lung, Lymphoma |
| 38 | CD63 | CD63 | Lung |
| 39 | CD24 | CD24 | Hepatocellular |
| 40 | IL2RB | IL2RB | Colorectal |
| 41 | Lymphocite antigen 9 | LY9 | Prostate |
| 42 | complement factor B | CFAB | Ovarian |
| 43 | NY-ESO-1 | NY-ESO-1 | Lung |
| 44 | CAGE | CAGE | Lung |
| 45 | GBU4-5 | GBU4-5 | Lung |
| 46 | SOX2 | SOX2 | Lung |
| 47 | HuD | HuD | Lung |
| 48 | IgM autoantibodies | IgM autoantibodies | Lung |
| 49 | peroxiredoxin VI | Prx VI | Oesophageal |
| 50 | Anti-BMI1 polycomb ring finger oncogene | Bmi-1 | Oesophageal |
| 51 | human forkhead/winged helix transcription factor | FOXP3 | Oesophageal |
| 52 | Anti-CTDSP1 (carboxy-terminal domain, RNA polymerase II, polypeptide A, small phosphatase 1) | CTDSP1 | Pancreatic |
| 53 | Anti-MAPK9 (mitogen-activated protein kinase 9) | MAPK9 | Pancreatic |
| 54 | Anti-NR2E3 (nuclear receptor 2E3) | NR2E3 | Pancreatic |
| 55 | Anti-hydroxysteroid-(17-alpha)-dehydrogenase |  | NSCLC |
| 56 | Anti-triosephosphate isomerase |  | NSCLC |
| 57 | complement component C3a | complement component C3a | Breast |
