# Supplementary material for: Building the Evidence Base of Blood-Based Biomarkers for Early Detection of Cancer: A Rapid Systematic Mapping Review
Source: eBioMedicine. 2016 Jul 6;10:164–73. doi: 10.1016/j.ebiom.2016.07.004 (PMC5006664; doi:10.1016/j.ebiom.2016.07.004)
Supplement: Supplementary Table 3 — Classical tumour markers. [file mmc3.docx]

**Supplementary Table 3: Classical Tumour Markers**

| No | **Biomarker** | **Acronym** | **Cancer** |
| --- | --- | --- | --- |
| 1 | Prostate specific antigen | PSA; PSA alpha1-antichymotrypsin; prostate-specific antigen velocity per initial volume; ProPSA | Prostate |
| 2 | cancer antigen 15-3 | CA15-3; CA 15-3 | Breast, Endometrial, Lung, Ovarian |
| 3 | carcinoembryonic antigen | CEA | Bladder, Breast, Colorectal, Gastric, General, Hepatocellular, Lung |
| 4 | alpha-fetoprotein | AFP, AFP-L3 = Lens culinaris agglutinin-reactive AFP | General, Hepatocellular, Testicular |
| 5 | lactate dehydrogenase | LDH | Breast |
| 6 | Human epididymis protein 4 | HE4 | Endometrial, Lung, Ovarian |
| 7 | Carbohydrate antigen 125 | CA125; CA-125, ROMA = risk of ovarian malignancy algorithm | Breast, Endometrial, General, Hepatocellular, Mesothelioma, Ovarian |
| 8 | Carbohydrate antigen 19-9 | CA19-9; CA199 | Bilary tract, Colorectal, Gastrointestinal, Pancreatic |
| 9 | squamous cell carcinoma antigen | SCCA; SCC-ag | Cervical, Hepatocellular, Lung |
| 10 | Carbonic anhydrase | CA9 | Renal |
| 11 | cytokeratin fragment 19 | CYFRA 21-1 | Lung, Mesothelioma, Pancreatic |
| 12 | Neuron Specific Enolase | NSE | Lung, Renal |
| 13 | leptin | leptin | Breast, Prostate |
| 14 | Progastrin-releasing peptide | proGRP | Lung |
| 15 | Cancer antigen 72-4 | CA72-4 | Gastrointestinal, Ovarian |
| 16 | tyrosinase | tyrosinase | Melanoma |
| 17 | squamous cell carcinoma antigen-immunoglobulin M | SCCA-IgM | Hepatocellular |
| 18 | PCA3 | PCA3 | Prostate |
| 19 | Adenosine deaminase | ADA | Breast |
| 20 | Gamma-glutamyl transferase | GGT; gamma-glutamyl transpeptidase | Breast |
| 21 | Alkaline phosphatase | ALP | Breast |
| 22 | HER2 | HER2; AB_HER2; 36 HER2 negative; erbb-2; soluble human epidermal growth factor receptor 2 (sHER2) | Breast, Lung |
| 23 | PSMA | PSMA | Prostate |
