# Supplementary material for: Building the Evidence Base of Blood-Based Biomarkers for Early Detection of Cancer: A Rapid Systematic Mapping Review
Source: eBioMedicine. 2016 Jul 6;10:164–73. doi: 10.1016/j.ebiom.2016.07.004 (PMC5006664; doi:10.1016/j.ebiom.2016.07.004)
Supplement: Supplementary Table 4 — Coagulation and angiogenesis molecules. [file mmc4.docx]

**Supplementary Table 4: Coagulation and Angiogenesis Molecules**

| **No** | **Biomarker** | **Acronym** | **Cancer** |
| --- | --- | --- | --- |
| 1 | urokinase plasminogen activator | uPA/uPAR/ suPAR | Lung, Prostate |
| 2 | vascular endothelial growth factor | VEGF | Colorectal, General, Lung, Ovarian |
| 3 | Annexin A4 | ANXA4 | General |
| 4 | hemoglobin-alpha | Hb-alpha | Ovarian |
| 5 | hemoglobin-beta | Hb-beta | Ovarian |
| 6 | Des-gamma-carboxyprothrombin | DCP | General, Hepatocellular |
| 7 | Kallikrein 10 | KLK10 | Uterine |
| 8 | kininogen-1 | kininogen-1 | Colorectal, Hepatocellular |
| 9 | Kallikrein 4 | KLK4 | Prostate |
| 10 | Endothelin-1 | ET-1 | Lung |
| 11 | plasminogen activator inhibitor-1 | PAI-1 | Breast, Ovarian |
| 12 | Kallikrein 6 | KLK6 | Ovarian |
| 13 | Angiopoietin-2 | Angiopoietin-2; Apo-2 | Lung |
| 14 | thrombospondin-1 | THBS1 | Breast, Endometrial, Lung |
| 15 | plasminogen activator inhibitor | plasminogen activator inhibitor | Lung |
| 16 | Protease Activated Receptor | PAR1; PAR2 | Breast |
| 17 | coagulation factor V | coagulation factor V | Prostate |
| 18 | des-- carboxy prothrombin, 2-9 Glu residues ratio | DCP, NX-PVKA ratio | Hepatocellular |
| 19 | endostatin | endostatin | Lung |
| 20 | Placental growth factor | PlGF | Endometrial |
| 21 | Annexin A1 | ANXA1 mNRA | Lung, Oral |
| 22 | Nestin | Nestin | Melanoma |
| 23 | Endothelial cell-specific molecule-1 | ESM-1 | Colorectal, Hepatocellular |
| 24 | C4d | C4d | Lung |
| 25 | annexin A2 | ANXA2 | Hepatocellular, Lung |
| 26 | Pigment Epithelium Derived Factor | PEDF | Hepatocellular |
| 27 | thrombomodulin | THBD-M | Colorectal |
| 28 | Annexin A3 | ANXA3 | Colorectal |
| 29 | Von Willebrand factor | VWF | Hepatocellular |
