# Supplementary material for: Building the Evidence Base of Blood-Based Biomarkers for Early Detection of Cancer: A Rapid Systematic Mapping Review
Source: eBioMedicine. 2016 Jul 6;10:164–73. doi: 10.1016/j.ebiom.2016.07.004 (PMC5006664; doi:10.1016/j.ebiom.2016.07.004)
Supplement: Supplementary Table 5 — Cytokines, chemokines and insulin-like growth factors. [file mmc5.docx]

**Supplementary Table 5: Cytokines, Chemokines and Insulin-like Growth Factors**

| **No** | **Biomarker** | **Acronym** | **Cancer** |
| --- | --- | --- | --- |
| 1 | interleukin 2 | IL-2 | General, Ovarian |
| 2 | interleukin 6 | IL-6 | Breast, General, Oral, Ovarian |
| 3 | interleukin 8 | IL-8 | Breast, Colorectal, General, Oral, Ovarian, Prostate |
| 4 | interleukin 10 | IL-10 | General |
| 5 | interleukin 12 | IL-12 | General, Ovarian |
| 6 | interleukin 18 | IL-18 | General, Hepatocellular |
| 7 | tumour necrosis factor [alpha] | TNF[alpha]; DcR3 | Breast, General, Lung, Ovarian |
| 8 | transforming growth factor [beta] | TGF[beta]/ TGF-beta-1 | Breast, General, Hepatocellular |
| 9 | interferon-[gamma] | interferon-[gamma] | General |
| 10 | macrophage migration inhibitory factor | MIF | General, Lung |
| 11 | C-X-C motif chemokine receptor 4 | CXCR4 | General |
| 12 | human glandular kallikrein 2 | hK2 | Prostate |
| 13 | macrophage inhibitory cytokine-1 | MIC-1 | Breast, Pancreatic |
| 14 | macrophage-colony stimulating factor | MCSF; GM-CSF | Breast, Cervical, Endometrial, Oral, Ovarian, Pancreatic |
| 15 | Golgi protein 73 | GP73 | Hepatocellular |
| 16 | epidermal growth factor receptor | EGFR; C-erbB-2 | Cervical, Gastric, Hepatocellular |
| 17 | Insulin-like growth factor-binding protein-2 | IGFBP-2 | Colorectal |
| 18 | hepatocyte growth factor | HGF | Breast, Lung |
| 19 | insulin-like growth factor binding protein | IGFBP-3 | General, Lung, Pancreatic |
| 20 | Granulocyte-colony stimulating factor | G-CSF | Lung |
| 21 | interleukin 3 | IL-3 | Lung |
| 22 | stem cell factor | SCF | Endometrial, Lung |
| 23 | insulin-like growth factor | IGF-I | General, Prostate |
| 24 | Insulin growth factor blood protein 1 | IGFBP1 | Ovarian |
| 25 | C-C motif chemokine 5 | C-C motif chemokine 5 | Lung |
| 26 | brain-derived neurotrophic factor | BDNF | Colorectal |
| 27 | Chemokine (C-X-C Motif) Ligand-1 | CXCL1 | Breast, Ovarian, Prostate |
| 28 | interleukin-1ra | IL-1ra | Colorectal, Lung |
| 29 | monocyte chemotactic protein-1 | MCP-1 | Hepatocellular, Lung |
| 30 | chemokine (C-X-C motif) ligand 9 | CXCL9 | Prostate |
| 31 | Midkine | MK; MDK | Hepatocellular, Lung, Ovarian |
| 32 | Interleukin-33 | IL-33 | Gastric |
| 33 | growth differentiation factor-15 | GDF-15 | Uterine |
| 34 | CC chemokine ligand 11 | CCL11 | Ovarian |
| 35 | CC chemokine ligand 18 | CCL18 | Ovarian |
| 36 | interleukin 16 | il-16 | Myeloma |
| 37 | regulated upon activation, normally T-expressed and presumably secreted | RANTES | Breast, Gastric |
| 38 | IRF1 | IRF1 | Lung |
| 39 | soluble tumor necrosis factor-alpha receptors 1 | sTNFR1 | Prostate |
| 40 | interleukin 4 | IL-4 | Ovarian |
| 41 | interleukin 13 | IL-13 | Ovarian |
| 42 | CXCL13 | CXCL13 | Lymphoma |
| 43 | transforming growth factor alpha | TGF-alpha | Hepatocellular |
| 44 | Chemokine-10 | CXCL10 | Glioma |
| 45 | interleukin-27 | IL-27 | Hepatocellular |
| 46 | macrophage stimulating protein alpha | MSP-alpha | Ovarian |
| 47 | platelet-derived growth factor receptor alpha | PDGF-R alpha | Ovarian |
| 48 | Heparin-binding EGF-like growth factor | HB-EGF | Ovarian |
| 49 | nervous growth factor | NGF | Hepatocellular |
| 50 | TNFAIP6 | TNFAIP6 | Colorectal |
| 51 | Macrophage inflammatory protein 4 | MIP-4 | Lung |
| 52 | megakaryocyte potentiating factor | MPF | Mesothelioma |
