# Supplementary material for: Building the Evidence Base of Blood-Based Biomarkers for Early Detection of Cancer: A Rapid Systematic Mapping Review
Source: eBioMedicine. 2016 Jul 6;10:164–73. doi: 10.1016/j.ebiom.2016.07.004 (PMC5006664; doi:10.1016/j.ebiom.2016.07.004)
Supplement: Supplementary Table 6 — Circulating-free DNA. [file mmc6.docx]

**Supplementary Table 6: Circulating-free DNA**

| **No** | **Biomarker** | **Acronym** | **Cancer** |
| --- | --- | --- | --- |
| 1 | Microsatellite alterations at FHIT | FHIT | Lung |
| 2 | microsatellite alterations at loci on chromosome 3 | 3p loci | Lung |
| 3 | adenomatous polyposis coli | APC | Lung, CRC, Renal |
| 4 | CHD1 | CHD1 | Lung |
| 5 | O(6)-methyl-guanine-DNA methyltransferase | MGMT | Lung |
| 6 | DCC | DCC | Lung |
| 7 | RASSF1A | RASSF1A | Breast, Hepatocellular, Lung, Oesophageal |
| 8 | absent in melanoma 1 | AIM1; Beta/gamma crystallin domain-containing protein 1 | Lung |
| 9 | Septin 9 | Septin 9 | Colorectal |
| 10 | BRCA1 associated RING domain 1 FL | BARD1 FL | Breast |
| 11 | BRCA1 associated RING domain 1 delta | BARD1 delta | Breast |
| 12 | BRCA1 associated RING domain 1 AKIN | BARD1 AKIN | Breast |
| 13 | mitochondrial DNA | mtDNA | Breast, General |
| 14 | IgH | FR3A/VLJH | Lymphoma |
| 15 | TCR gamma | TVG/TJX | Lymphoma |
| 16 | GSTP1 | GSTP1 | Breast, Prostate |
| 17 | methylation of CYCD2 | CYCD2 | Colorectal |
| 18 | methylation of HIC1 | HIC1 | Colorectal |
| 19 | methylation of PAX 1 | PAX 1 | Colorectal |
| 20 | methylation of RB1 | RB1 | Colorectal |
| 21 | methylation of SRBC | SRBC | Colorectal |
| 22 | FAM5C | FAM5C | Gastric |
| 23 | MYLK | MYLK | Gastric |
| 24 | glyceraldehyde-3-phosphate dehydrogenase | GAPDH | Breast |
| 25 | Retinoid-acid-receptor-beta gene | RAR-B | Oesophageal, Renal |
| 26 | ARF tumor suppressor protein gene | p14(ARF) | Renal |
| 27 | Prostaglandin-endoperoxid synthase 2 | PTGS2 | Renal |
| 28 | Gluthation-a-transferase-protein 1 gene | PTGS2 | Renal |
| 29 | SLC5A8 hypermethylation | SLC5A8 SLC26A4 hypermethylation | Thyroid |
| 30 | SLC26A4 hypermethylation | SLC26A4 hypermethylation | Thyroid |
| 31 | BRAF (V600E) | BRAF (V600E) | Thyroid |
| 32 | RARbeta2 | RARbeta2 | Breast |
| 33 | human telomerase reverse transcriptase DNA | hTERT DNA | Hepatocellular |
| 34 | Line1 79 bp | Line1 79 bp | Colorectal |
| 35 | Line1 300 bp | Line1 300 bp | Colorectal |
| 36 | Alu 115 bp | Alu 115 bp | Colorectal |
| 37 | Alu 247 bp | Alu 247 bp | Colorectal |
| 38 | basonuclin 1 | BNC1 | Pancreatic |
| 39 | ADAM metallopeptidase with thrombospondin type 1 motif, 1 | ADAMTS1 | Pancreatic |
