# Supplementary material for: Building the Evidence Base of Blood-Based Biomarkers for Early Detection of Cancer: A Rapid Systematic Mapping Review
Source: eBioMedicine. 2016 Jul 6;10:164–73. doi: 10.1016/j.ebiom.2016.07.004 (PMC5006664; doi:10.1016/j.ebiom.2016.07.004)
Supplement: Supplementary Table 7 — Hormones. [file mmc7.docx]

**Supplementary Table 7: Hormones**

| **No** | **Biomarker** | **Acronym** | **Cancer** |
| --- | --- | --- | --- |
| 1 | human chorionic gonadotropin | hCG | Breast |
| 2 | gastrin-17 | G-17 | Gastric |
| 3 | plasma seratonin 5-hydroxytryptamine | plasma seratonin 5-hydroxytryptamine | Breast |
| 4 | retinoic acid receptor beta | RAR beta | Breast, Oesophageal |
| 5 | estradiol (2) | E(2) | Cervical |
| 6 | free testeosterone | free testeosterone | Cervical |
| 7 | High 25-hydroxyvitamin D | 25(OH)D | Breast |
| 8 | hypothalamic pituitary adrenal axis | HPAA | Gastric |
| 9 | progesterone receptor B | PRB | Lung |
| 10 | Corticosteroid-binding globulin | CBG | Ovarian |
| 11 | Follicle-stimulating hormone | FSH | Endometrial |
| 12 | calcitonin | calcitonin | Thyroid |
| 13 | prolactin | prolactin | Breast, Endometrial, Lung |
