# Supplementary material for: Building the Evidence Base of Blood-Based Biomarkers for Early Detection of Cancer: A Rapid Systematic Mapping Review
Source: eBioMedicine. 2016 Jul 6;10:164–73. doi: 10.1016/j.ebiom.2016.07.004 (PMC5006664; doi:10.1016/j.ebiom.2016.07.004)
Supplement: Supplementary Table 8 — Metabolic markers. [file mmc8.docx]

**Supplementary Table 8: Metabolic markers**

| **No** | **Biomarker** | **Acronym** | **Cancer** |
| --- | --- | --- | --- |
| 1 | plasma glucose levels | plasma glucose levels | Cervical, Colorectal, Pancreatic |
| 2 | Serum 27-nor-5beta-cholestane-3,7,12,24,25 pentol glucuronide | CPG | Ovarian |
| 3 | malonic acid | malonic acid | Gastrointestinal |
| 4 | L-serine | L-serine | Oesophageal |
| 5 | 3-hydroxypropionic acid and pyruvic acid | 3-hydroxypropionic acid and pyruvic acid | Colorectal, Gastric |
| 6 | alanine | L-alanine, glucuronoic lactone | Colorectal, Lung |
| 7 | L-glutamine | glutamine | Colorectal |
| 8 | sarcosine | sarcosine | Colorectal, Prostate |
| 9 | phosphatidylserine | phosphatidylserine | Oesophageal |
| 10 | phosphatidic acid | phosphatidic acid | Oesophageal |
| 11 | choline | phosphatidyl choline; (PC) (34 : 1) | Colorectal, Gastric, Hepatocellular, Oesophageal |
| 12 | phosphatidylinositol | phosphatidylinositol | Colorectal, Oesophageal |
| 13 | phosphatidyl ethanolamine | phosphatidyl ethanolamine | Oesophageal |
| 14 | sphinganine 1-phosphate | S-1-P | Hepatocellular, Oesophageal |
| 15 | carnitine | carnitine | Myeloma |
| 16 | acetylcarnitine | acetylcarnitine | Myeloma |
| 17 | L-valine | valine | Colorectal |
| 18 | L-threonine | threonine | Colorectal |
| 19 | 1-deoxyglucose | 1-deoxyglucose | Colorectal |
| 20 | glycine | glycine | Colorectal |
| 21 | MACF1 | MACF1 | Colorectal |
| 22 | apolipoprotein H | APOH; beta-2-glycoprotein | Colorectal |
| 23 | alpha-2-macroglobulin | A2M | Colorectal |
| 24 | Immunoglobulin lambda locus | IGL@ | Colorectal |
| 25 | vitamin D-binding protein | VDB | Colorectal |
| 26 | leucine | leucine; isoleucine | Lung |
| 27 | histidine | histidine | Lung |
| 28 | tryptophan | tryptophan | Lung |
| 29 | ornithine | ornithine | Lung |
| 30 | 2-hydroxyglutarate | 2-hydroxyglutarate | Colorectal |
| 31 | glutamate | glutamate | Hepatocellular |
| 32 | acetate | acetate | Hepatocellular |
| 33 | N-acetyl glycoproteins | N-acetyl glycoproteins | Hepatocellular |
| 34 | 2-hydroxybutyrate | 2-hydroxybutyrate | Colorectal |
| 35 | aspartic acid | aspartic acid | Colorectal |
| 36 | kynurenine | kynurenine | Colorectal |
| 37 | cystamine | cystamine | Colorectal |
| 38 | Lactic acid | Lactic acid | Lung |
| 39 | Glycelic acid | Glycelic acid | Lung |
| 40 | Glycolic acid | Glycolic acid | Lung, Oesophageal |
| 41 | lysophosphatidylcholine | lysoPC a C16:0; lysoPC(16:1); lysoPC 17:0 | Breast, Hepatocellular, Oesophageal |
| 42 | PC ae C42:5 | PC ae C42:5 | Breast |
| 43 | PC aa C34:2 | PC aa C34:2 | Breast |
| 44 | glycochenodeoxycholic acid 3-sulfate | 3-sulfo-GCDCA | Hepatocellular |
| 45 | glycocholic acid | GCA | Hepatocellular |
| 46 | glycodeoxycholic acid | GDCA | Hepatocellular |
| 47 | taurocholic acid | TCA | Hepatocellular |
| 48 | taurochenodeoxycholate | TCDCA | Hepatocellular |
| 49 | alpha-glutamyl dipeptides | ?-glutamyl dipeptides | Hepatocellular |
| 50 | tricarboxylic acid | TCA | Colorectal, Oesophageal |
| 51 | DUPAN-2 | DUPAN-2 | Pancreatic |
| 52 | SPAN-1 | SPAN-1 | Pancreatic |
| 53 | 2-aminoethanesulfonic acid | taurine | Colorectal |
| 54 | lactate | lactate | Colorectal |
| 55 | phosphocholine | phosphocholine | Colorectal |
| 56 | proline | proline | Colorectal |
| 57 | phenylalanine | phenylalanine | Colorectal |
| 58 | 3beta | 3beta | Hepatocellular |
| 59 | 6beta-dihydroxy-5beta-cholan-24-oic acid | 6beta-dihydroxy-5beta-cholan-24-oic acid | Hepatocellular |
| 60 | oleoyl carnitine | oleoyl carnitine | Hepatocellular |
| 61 | Phe-Phe | Phe-Phe | Hepatocellular |
| 62 | octanoylcarnitine | octanoylcarnitine | Oesophageal |
| 63 | decanoylcarnitine | decanoylcarnitine | Oesophageal |
| 64 | lipid metabolism | lipid metabolism | Oesophageal |
| 65 | amino acid metabolism | amino acid metabolism | Oesophageal |
| 66 | ketogenesis | ketogenesis | Oesophageal |
| 67 | energy metabolism | energy metabolism | Oesophageal |
| 68 | C-18:1 | C-18:1 | Pancreatic |
| 69 | C-18:2 | C-18:2 | Pancreatic |
| 70 | C-18:3 | C-18:3 | Pancreatic |
| 71 | C-18:2/C-18:1 | C-18:2/C-18:1 | Pancreatic |
| 72 | C-18:3/C-18:1 | C-18:3/C-18:1 | Pancreatic |
| 73 | C-20:4 | C-20:4 | Pancreatic |
| 74 | C-22:6 | C-22:6 | Pancreatic |
| 75 | Palmitoylcarnitine | Palmitoylcarnitine | Gastric |
| 76 | m/z 361.2346 | m/z 361.2346 | Gastric |
| 77 | Total Sialic Acid | TSA | Oral |
| 78 | Lipid-Bound Sialic Acid | LSA | Oral |
| 79 | 1-methyladenosine | 1-methyladenosine | Oesophageal |
| 80 | N(2)-dimethylguanosine | N(2)-dimethylguanosine | Oesophageal |
| 81 | N(2)-methylguanosine | N(2)-methylguanosine | Oesophageal |
| 82 | cytidine | cytidine | Oesophageal |
| 83 | N-linked glycan levels | N-glycans | Breast, Ovarian, Renal |
| 84 | Breast cancer 1 | Bc1 | Breast |
| 85 | Breast cancer 2 | Bc2 | Breast |
| 86 | Breast cancer 3 | Bc3 | Breast |
| 87 | NG1A2F | NG1A2F | Lung |
| 88 | methionine | methionine | Hepatocellular |
| 89 | N-glycopeptides | glycopeptides | Mesothelioma |
| 90 | plasma C9 protein | C9 | Gastric |
| 91 | uric acid | UA | Pancreatic |
| 92 | Apolipoprotein C-III | apoC-III | Gastric |
| 93 | vitamin B12 | cobalamin | Prostate |
| 94 | folate | folate | Prostate |
| 95 | N-glycosylated protein | N-glycoprotein | Lymphoma |
| 96 | transferrin | TF | Endometrial, Oral |
| 97 | myeloperoxidase | MPO | Ovarian |
| 98 | free iron | free iron | Ovarian |
| 99 | lipid hydro peroxides | LOOH | Cervical, Ovarian |
| 100 | ALU-like | ALU-like | Hepatocellular |
| 101 | mammalian target of rapamycin | mTOR | Cervical |
| 102 | oleamide | oleamide | Colorectal, Lung |
| 103 | long chain acyl carnitines | long chain acyl carnitines | Lung |
| 104 | lysophosphatidylcholine 18:1 | LPC(18:1) | Lung |
| 105 | lysophosphatidylcholine 20:4 | LPC(20:4) | Lung |
| 106 | lysophosphatidylcholine 20:3 | LPC(20:3) | Lung |
| 107 | lysophosphatidylcholine 22:6 | LPC(22:6) | Lung |
| 108 | serum metabolite 16:0/1 | SM(16:0/1) | Lung |
| 109 | total cholesterol | T-C | Breast, Prostate |
| 110 | high density lipoprotein cholesterol | HDL-C | Breast, Prostate |
| 111 | leukocyte methylated cytosine 5 | 5-mC | Colorectal |
| 112 | Fatty acid synthase | FAS | Gastric |
| 113 | O-linked glycans | O-linked glycans | Ovarian |
| 114 | glycosphingolipids | glycosphingolipids | Ovarian |
| 115 | ferritin | FTL | Lung, Renal |
| 116 | Plasma choline-containing phospholipids | Plasma phospholipids | Colorectal |
| 117 | 7762 Da^[[1]](#footnote-1)^ | 7762 Da | Pancreatic |
| 118 | 8560 Da^1^ | 8560 Da | Pancreatic |
| 119 | 11654 Da^1^ | 11654 Da | Pancreatic |
| 120 | palmitic amide | palmitic amide | Colorectal |
| 121 | hexadecanedioic acid | hexadecanedioic acid | Colorectal |
| 122 | octadecanoic acid | octadecanoic acid | Colorectal |
| 123 | eicosatrienoic acid | eicosatrienoic acid | Colorectal |
| 124 | lysophosphatidylcholine 18:2 | LPC(18:2) | Colorectal |
| 125 | lysophosphatidylcholine 16:0 | LPC(16:0) | Colorectal |
| 126 | 5-hydroxyindoleacetic acid | 5-HIAA | Neuroendocrine |

1. Qian JY, Mou SH, Liu CB. SELDI-TOF MS combined with magnetic beads for detecting serum protein biomarkers and establishment of a boosting decision tree model for diagnosis of pancreatic cancer. Asian Pacific Journal of Cancer Prevention. 2012;13(5):1911-5. [↑](#footnote-ref-1)
