# Supplementary material for: Building the Evidence Base of Blood-Based Biomarkers for Early Detection of Cancer: A Rapid Systematic Mapping Review
Source: eBioMedicine. 2016 Jul 6;10:164–73. doi: 10.1016/j.ebiom.2016.07.004 (PMC5006664; doi:10.1016/j.ebiom.2016.07.004)
Supplement: Supplementary Table 9 — MicroRNA and other RNAs. [file mmc9.docx]

**Supplementary Table 9: MicroRNA and other RNAs**

| **No** | **Biomarker** | **Acronym** | **Cancer** |
| --- | --- | --- | --- |
| 1 | let-7a | let-7a | Breast, Ovarian, Prostate |
| 2 | let-7c | let-7c; has-let-7c | Bladder, Prostate, Renal |
| 3 | let-7e | let-7e; let-7e* | Ovarian, Retinoblastoma |
| 4 | let-7f | let-7f | Ovarian |
| 5 | let-7g |  | Colorectal cancer |
| 6 | miR-101 | miR-101 | Hepatocellular, prostate, breast cancer |
| 7 | miR-103 | miR-103 | Mesothelioma, Ovarian |
| 8 | miR-106a | miR-106a | Breast |
| 9 | miR-106b | miR-106b | Leukemia |
| 10 | miR-10b | miR-10b | Breast, Oral |
| 11 | miR-122 | miR-122 | Hepatocellular, Pancreatic |
| 12 | miR-1246 | miR-1246 | Cervical, Myeloma, Ovarian |
| 13 | miR-1253 | miR-1253 | Oral |
| 14 | miR-1254 | miR-1254 | Lung |
| 15 | miR-126 | miR-126 | Colorectal, Mesothelioma, Oesophageal, pancreatic |
| 16 | miR-1260 | miR-1260 | Breast |
| 17 | miR-127-3p | miR-127-3p | Breast |
| 18 | miR-1280 | miR-1280 | Breast |
| 19 | miR-1283 | miR-1283 | Oral |
| 20 | miR-128b | miR-128b | Leukemia, Lung |
| 21 | miR-129 | miR-129 | Leukemia |
| 22 | miR-129-2 | miR-129-2 | Hepatocellular |
| 23 | miR-1290 | miR-1290 | Ovarian |
| 24 | miR-1303 | miR-1303 | Larygeal |
| 25 | miR-1308 | miR-1308 | Myeloma |
| 26 | miR-130b | miR-130b | Hepatocellular, Ovarian |
| 27 | miR-132 | miR-132 | Ovarian |
| 28 | miR-1322 | miR-1322 | Oesophageal |
| 29 | miR-133a | miR-133a | Bladder, Lung, Oesophageal, Prostate, Renal |
| 30 | miR-133b | miR-133b | Oesophageal |
| 31 | miR-135a | miR-135a | Leukemia |
| 32 | miR-135b | miR-135b | Colorectal |
| 33 | miR-136 | miR-136 | Bladder, Prostate, Renal |
| 34 | miR-139-5p | miR-139-5p | Melanoma |
| 35 | miR-140 | miR-140 | Lung |
| 36 | miR-141 | miR-141 | Colorectal, Ovarian, Prostate |
| 37 | miR-142-5p | miR-142-5p | Ovarian |
| 38 | miR-143 | miR-143 | Colorectal, Lung, Oesophageal |
| 39 | miR-145 | miR-145 | Bladder, Breast, Colorectal, Melanoma, Oesophageal, Ovarian, Pancreatic, Prostate, Renal |
| 40 | miR-145-5p | miR-145-5p | Oral |
| 41 | miR-1468 | miR-1468 | Lung |
| 42 | miR-146a | miR-146a | Breast, Melanoma |
| 43 | miR-146b-3p | miR-146b-3p | Lung |
| 44 | miR-148b | miR-148b | Breast, Leukemia |
| 45 | miR-150 | miR-150 | Hepatocellular, Leukemia, Melanoma, Pancreatic |
| 46 | miR-150-5p | miR-150-5p | Oral |
| 47 | miR-151-3p | miR-151-3p | Melanoma |
| 48 | miR-151-5p | miR-151-5p | Gastric |
| 49 | miR-153 | miR-153 | Leukemia |
| 50 | miR-155 | miR-155 | Breast, Lung, Lymphoma, Melanoma, Nasopharyngeal carcinoma, Ovarian, Prostate |
| 51 | miR-159a | miR-159a | Leukemia |
| 52 | miR-15a | miR-15a | Lymphoma, Oesophageal |
| 53 | miR-15b | miR-15b | Hepatocellular, Lung |
| 54 | miR-16 | miR-16 | Bilary tract, Breast, Nasopharyngeal carcinoma, Pancreatic, Prostate |
| 55 | miR-16-1 | miR-16-1 | Lymphoma |
| 56 | miR-17 | miR-17 | General |
| 57 | miR-17-3p | miR-17-3p | Colorectal |
| 58 | miR-17-5p | miR-17-5p | Melanoma |
| 59 | miR-181a | miR-181a | Breast, Leukemia |
| 60 | miR-181c | miR-181c | Breast, Leukemia, Lung |
| 61 | miR-182 | miR-182 | Breast, Lung |
| 62 | miR-183 | miR-183 | Leukemia |
| 63 | miR-184 | miR-184 | Leukemia, Ovarian |
| 64 | miR-185 | miR-185 | Bilary tract |
| 65 | miR-186 | miR-186 | Endometrial |
| 66 | miR-187 | miR-187 | Bladder, Leukemia, Prostate, Renal |
| 67 | miR-188-3p | miR-188-3p | Leukemia |
| 68 | miR-18a | miR-18a | Colorectal, General, Hepatocellular, Leukemia, Lung, Oesophageal |
| 69 | miR-190 | miR-190 | Ovarian |
| 70 | miR-191 | miR-191 | Breast, Melanoma, Renal |
| 71 | miR-191-5p | miR-191-5p | Colorectal |
| 72 | miR-192 | miR-192 | Hepatocellular, Leukemia |
| 73 | miR-193a-3p | miR-193a-3p | Leukemia |
| 74 | miR-193a-5p | miR-193a-5p | Leukemia |
| 75 | miR-193b* | miR-193b* | Ovarian |
| 76 | miR-195 | miR-195 | Breast, Prostate |
| 77 | miR-195-5p | miR-195-5p | Gastric |
| 78 | miR-196a | miR-196a | Oesophageal, Pancreatic |
| 79 | miR-196a2 | miR-196a2 | Bilary tract |
| 80 | miR-197 | miR-197 | Lung, Myeloma |
| 81 | miR-1973 | miR-1973 | Lymphoma |
| 82 | miR-198 | miR-198 | Renal |
| 83 | miR-199a-3p | miR-199a-3p | Gastric |
| 84 | miR-199a-5p | miR-199a-5p | Oral |
| 85 | miR-199b | miR-199b | Leukemia |
| 86 | miR-200a | miR-200a | Bladder, Breast, Ovarian, Prostate, Renal |
| 87 | miR-200b | miR-200b | Bladder, Breast, Leukemia, Ovarian, Prostate, Renal |
| 88 | miR-200c | miR-200c | Leukemia, Ovarian, Renal |
| 89 | miR-200c/141 | miR-200c/141 | Breast |
| 90 | miR-202 | miR-202 | Leukemia, Renal |
| 91 | miR-203 | miR-203 | Leukemia, Melanoma, Oral |
| 92 | miR-204 | miR-204 | Endometrial |
| 93 | miR-205 | miR-205 | Breast, Melanoma, Ovarian |
| 94 | miR-20a | miR-20a | Cervical, Colorectal, General, Leukemia |
| 95 | miR-21 | miR-21 | Bilary tract, Breast, Colorectal, Hepatocellular, Lung, Nasopharyngeal carcinoma, Oesophageal, Pancreatic, Prostate, Retinoblastoma |
| 96 | miR-210 | miR-210 | Pancreatic, Renal |
| 97 | miR-211 | miR-211 | Leukemia |
| 98 | miR-212 | miR212 | Lung, Prostate |
| 99 | miR-212- 3p | miR-212- 3p | Larygeal |
| 100 | miR-214 | miR-214 | Leukemia |
| 101 | miR-215 | miR-215 | Breast |
| 102 | miR-216 | miR-216 | Leukemia |
| 103 | miR-218 | miR-218 | Leukemia |
| 104 | miR-219 | miR-219 | Leukemia |
| 105 | miR-22 | miR-22 | Bilary tract |
| 106 | miR-220 | miR-220 | Leukemia, Lung |
| 107 | miR-220c | miR-220c | Leukemia |
| 108 | miR-221 | miR-221 | Gastric, Leukemia, Lung, Lymphoma, Melanoma, Renal |
| 109 | miR-222 | miR-222 | Breast, Endometrial, Renal |
| 110 | miR-223 | miR-223 | Endometrial, Hepatocellular, Leukemia, Oral, Pancreatic |
| 111 | miR-23a | miR-23a | Lung |
| 112 | miR-23a-3p | miR-23a-3p | Oral |
| 113 | miR-23b | miR-23b | Leukemia |
| 114 | miR-24 | miR-24 | Bilary tract, Nasopharyngeal carcinoma |
| 115 | miR-25 | miR-25 | Breast, Leukemia, Oesophageal, Renal |
| 116 | miR-25-3p | miR-25-3p | Bilary tract |
| 117 | miR-26a | miR-26a | Hepatocellular, Leukemia, Ovarian, Prostate |
| 118 | miR-26a-2* | miR-26a-2* | Renal |
| 119 | mir-26b | mir-26b | Pancreatic |
| 120 | miR-26b-5p | miR-26b-5p | Bladder |
| 121 | miR-27a | miR-27a | Hepatocellular |
| 122 | miR-27b | miR-27b | Leukemia, Lung, Oesophageal |
| 123 | miR-299-3p | miR-299-3p | Leukemia |
| 124 | miR-299-5p | miR-299-5p | Breast |
| 125 | miR-29a | miR-29a | Colorectal |
| 126 | miR-29c | miR-29c | Lymphoma, Renal |
| 127 | miR-301 | miR-301 | Leukemia |
| 128 | miR-302 | miR-302 | General |
| 129 | miR-302a | miR-302a | Testicular |
| 130 | miR-302b | miR-302b | Testicular |
| 131 | miR-302c | miR-302c | Leukemia, Testicular |
| 132 | miR-30a-5p | miR-30a-5p | Central nervous system |
| 133 | miR-30b | miR-30b | Melanoma |
| 134 | miR-30c | miR-30c | Leukemia, Melanoma |
| 135 | miR-30c-1* | miR-30c-1* | Lung |
| 136 | miR-31 | miR-31 | Bladder, Renal |
| 137 | miR-32 | miR-32 | Breast |
| 138 | miR-320 | miR-320 | Glioma, Leukemia, Retinoblastoma |
| 139 | miR-320c | miR-320c | Testicular |
| 140 | miR-324-3p | miR-324-3p | Breast, Leukemia |
| 141 | miR-324-5p | miR-324-5p | Ovarian |
| 142 | miR-328 | miR-328 | Leukemia |
| 143 | miR-329 | miR-329 | Leukemia |
| 144 | miR-33 | miR-33 | Bladder |
| 145 | miR-330 | miR-330 | Leukemia, Lung |
| 146 | miR-330-5p | miR-330-5p | Leukemia |
| 147 | miR-331 | miR-331 | Leukemia, Lung |
| 148 | miR-331-3p | miR-331-3p | Larygeal |
| 149 | miR-331-5p | miR-331-5p | Leukemia |
| 150 | miR-337-3p | miR-337-3p | Prostate, Renal |
| 151 | miR-339-3p | miR-339-3p | Leukemia |
| 152 | miR-339-5p | miR-339-5p | Lung |
| 153 | miR-340 | miR-340 | Leukemia |
| 154 | miR-342 | miR-342 | Breast, Leukemia |
| 155 | Mir-342-3p | Mir-342-3p | Leukemia, Oral, Prostate |
| 156 | miR-342-5p | miR-342-5p | Leukemia, Renal |
| 157 | miR-345 | miR-345 | Leukemia, Lung |
| 158 | miR-346 | miR-346 | Leukemia, Lung |
| 159 | miR-34a | miR-34a | Lymphoma, Pancreatic, Prostate |
| 160 | miR-34a* | miR-34a* | Ovarian |
| 161 | miR-361 | miR-361 | Leukemia |
| 162 | miR-362-3p | miR-362-3p | Leukemia |
| 163 | miR-363* | miR-363* | Ovarian |
| 164 | miR-368 | miR-368 | Bladder, Prostate, Renal |
| 165 | miR-369-3p | miR-369-3p | Leukemia |
| 166 | miR-371~373 | miR-371~373 | General, Testicular |
| 167 | miR-371a-3p | miR-371a-3p | Testicular |
| 168 | miR-372 | miR-372 | Testicular |
| 169 | miR-373 | miR-373 | Breast, Testicular |
| 170 | miR-375 | miR-375 | Oesophageal, Pancreatic |
| 171 | mir-376c | mir-376c | Gastric |
| 172 | miR-377 | miR-377 | Lung |
| 173 | miR-378 | miR-378 | Nasopharyngeal carcinoma, Renal |
| 174 | miR-409-3p | miR-409-3p | Breast |
| 175 | miR-411 | miR-411 | Breast |
| 176 | miR-423-5p | miR-423-5p | Oral |
| 177 | miR-451 | miR-451 | Bilary tract, Gastric, Leukemia, Oral, Renal |
| 178 | miR-452 | miR-452 | Breast |
| 179 | miR-483-5p | miR-483-5p | Hepatocellular, Leukemia, Myeloma, Ovarian |
| 180 | miR-484 | miR-484 | Leukemia, Lung |
| 181 | miR-492 | miR-492 | Bilary tract |
| 182 | miR-494 | miR-494 | Lymphoma |
| 183 | miR-505 | miR-505 | Pancreatic |
| 184 | miR-520c-3p | miR-520c-3p | Ovarian |
| 185 | miR-541 | miR-541 | Leukemia |
| 186 | miR-548-5p | miR-548-5p | Leukemia |
| 187 | miR-548as-3p | miR-548as-3p | Colorectal |
| 188 | miR-548b | miR-548b | Leukemia, Lung |
| 189 | miR-550 | miR-550 | Lung |
| 190 | miR-566 | miR-566 | Lung |
| 191 | miR-574-3p | miR574-3p | Glioma |
| 192 | miR-574-5p | miR-574-5p | Lung |
| 193 | miR-576-3p | miR-576-3p | Ovarian |
| 194 | miR-590-5p | miR-590-5p | Melanoma |
| 195 | miR-601 | miR-601 | Colorectal |
| 196 | miR-603 | miR-603 | Larygeal, Ovarian |
| 197 | miR-616* | miR-616* | Lung |
| 198 | miR-625* | miR-625* | Mesothelioma |
| 199 | mir-636 | mir-636 | Pancreatic |
| 200 | miR-645 | miR-645 | Ovarian |
| 201 | miR-652 | miR-652 | Breast |
| 202 | miR-655 | miR-655 | Leukemia |
| 203 | miR-656 | miR-656 | Lung |
| 204 | miR-660 | miR-660 | Leukemia, Lung |
| 205 | miR-660-5p | miR-660-5p | Larygeal |
| 206 | miR-661 | miR-661 | Central nervous system |
| 207 | miR-7 | miR-7 | Ovarian |
| 208 | miR-720 | miR-720 | Myeloma, Oral |
| 209 | miR-744 | miR-744 | Gastric |
| 210 | mir-760 | mir-760 | Colorectal |
| 211 | miR-769-3p | miR-769-3p | Leukemia |
| 212 | miR-801 | miR-801 | Breast, Hepatocellular |
| 213 | miR-876-3p | miR-876-3p | Lung |
| 214 | miR-885-5p | miR-885-5p | Colorectal, Pancreatic |
| 215 | miR-890 | miR-890 | Leukemia |
| 216 | miR-892a | miR-892a | Leukemia |
| 217 | miR-9 | miR-9 | Melanoma |
| 218 | miR-92 | miR-92 | Bladder, Lung, Ovarian |
| 219 | miR-92a | miR-92a | Colorectal, Leukemia |
| 220 | miR-93 | miR-93 | Renal |
| 221 | miR-939 | miR-939 | Lung |
| 222 | miR-99a | miR-99a | Oesophageal |
| 223 | miR-99b | miR-99b | Melanoma |
| 224 | miR-let-7 | let-7 | Lung |
| 225 | miR-let-7i | miR-let-7i | Prostate |
| 226 | miR-RNU44 | miR-RNU44 | Ovarian |
| 227 | miRNA-337-3p | miRNA-337-3p | Prostate |
| 228 | Piwi-interacting RNA-823 | piR-823 | Gastric |
| 229 | Piwi-interacting RNA651 | piR-651 | Gastric |
| 230 | snoRNA (RNU6) | snoRNA (RNU6) | Glioma |
| 231 | U6 snRNA (U6) | U6 snRNA (U6) | Colorectal |
| 232 | cell-free human telomerase reverse transcriptase (hTERT) mRNA | cell-free hTERT mRNA | Gastric, Prostate |
