# Supplementary material for: Building the Evidence Base of Blood-Based Biomarkers for Early Detection of Cancer: A Rapid Systematic Mapping Review
Source: eBioMedicine. 2016 Jul 6;10:164–73. doi: 10.1016/j.ebiom.2016.07.004 (PMC5006664; doi:10.1016/j.ebiom.2016.07.004)
Supplement: Supplementary Table 10 — Novel proteins. [file mmc10.docx]

**Supplementary Table 10: Novel Proteins**

| **No** | **Biomarker** | **Acronym** | **Cancer** |
| --- | --- | --- | --- |
| 1 | tissue polypeptide specific antigen | TPS | Pancreatic |
| 2 | tumor specific growth factor | TSGF | Pancreatic |
| 3 | Haptoglobin | HP | Lung, Pancreatic |
| 4 | Carbohydrate antigen 242 | CA 242 | Pancreatic |
| 5 | glypican-3 | GPC-3 | Hepatocellular |
| 6 | Melanoma-associated antigen 3 | MAGE-3; MAGE-A3 | Breast, Melanoma |
| 7 | Plasma filamin-A protein | Plasma filamin-A protein | Breast |
| 8 | cysteine-rich secretory protein 3 | CRISP-3 | Prostate |
| 9 | serum pepsinogen I (PGI pepsinogen I/II ratio | PGR | Gastric |
| 10 | 38559 Da | 38559 Da | Pancreatic |
| 11 | 9138 Da | 9138 Da | Pancreatic |
| 12 | 8925 Da | 8925 Da | Pancreatic |
| 13 | 3358 Da | 3358 Da | Pancreatic |
| 14 | paraoxonase 1 | PON1 | hepatocellular |
| 15 | Microtubule-associated protein RP/EB family member 1 | MAPRE1 | Colorectal |
| 16 | leucine-rich alpha-2-glycoprotein | LRG1 | Colorectal, Ovarian |
| 17 | neutrophil gelatinase-associated lipocalin | LCN2; NGAL | Pancreatic |
| 18 | Mycoplasma hyorhinis encoded protein p37 | p37 | Prostate |
| 19 | La-related protein 1 | LARP1 | Hepatocellular |
| 20 | UL16 binding protein 2 | ULBP2 | Pancreatic |
| 21 | CD9 | CD9 | Lung, Prostate |
| 22 | CD81 | CD81 | Lung, Prostate |
| 23 | REG4 | REG4 | Gastric, Pancreatic |
| 24 | Poly(rC)-binding protein-1 | PCBP-1 | Breast |
| 25 | Neural BC200 RNA | BC200 | Breast |
| 26 | tissue factor pathway inhibitor | TFPI | Pancreatic |
| 27 | Stathmin 1 | STMN1 | Bladder |
| 28 | carboxy-terminal domain, RNA polymerase II, polypeptide A, small phosphatase 1 | CTDSP1 | Pancreatic |
| 29 | EFNA1 | EFNA1 | Hepatocellular |
| 30 | EphA2 | EphA2 | Hepatocellular |
| 31 | CK20 | CK20 | Gastric |
| 32 | TFF1 | TFF1 | Gastric |
| 33 | Mucin 2 | MUC2 | Gastric |
| 34 | nectin 4 | nectin 4 | Ovarian |
| 35 | Fatty acid-binding protein-7 | FABP7 | Melanoma |
| 36 | stress-induced phosphoprotein-1 | STIP-1 | Ovarian |
| 37 | leptin/adiponectin (L/A) ratio | leptin/adiponectin (L/A) ratio | Breast |
| 38 | pro-apolipoprotein A1 | proApoA1 | Gastric |
| 39 | HMGA1 | HMGA1 | Lung |
| 40 | TFDP1 | TFDP1 | Lung |
| 41 | SUV39H1 | SUV39H1 | Lung |
| 42 | RBL1 | RBL1 | Lung |
| 43 | HNRPD | HNRPD | Lung |
| 44 | alpha-1-fucosidase | AFU | Hepatocellular |
| 45 | chromogranin A | CgA | Hepatocellular |
| 46 | lipopolysaccharide binding protein-1 | lipopolysaccharide binding protein-1 | Ovarian |
| 47 | proteoglycan-4 | proteoglycan-4 | Ovarian |
| 48 | pyridoxal-5-phosphate | PLP | Hepatocellular |
| 49 | hereditary prostate cancer 1 | HPC1; Gene ID: 6041 | Prostate |
| 50 | TMPRSS2 | TMPRSS2 | Prostate |
| 51 | ETS | ETS | Prostate |
| 52 | alpha-methylacyl-CoA racemase | AMACR | Prostate |
| 53 | GOLPH2 | GOLPH2 | Prostate |
| 54 | EPCA | EPCA | Prostate |
| 55 | serum leukocyte protease inhibitor | SLPI | Ovarian |
| 56 | Alpha-enolase | Alpha-enolase | Colorectal, Hepatocellular |
| 57 | high mobility group box-1 | HMBG1 | Pancreatic |
| 58 | anterior gradient 2 | AGR2 | Lung, Ovarian |
| 59 | Connective Tissue Activating Protein III | CTAPIII | Ovarian |
| 60 | Apolipoprotein J | Apo-J | Hepatocellular |
| 61 | Dickkopf-1 | DKK-1 | General |
| 62 | betaine | betaine | Colorectal |
| 63 | pentraxin-3 | PTX3 | Lung |
| 64 | ferritin heavy chain | FTH1 | Breast |
| 65 | alpha-2-heremans-schmid-glycoprotein | AHSG | Breast |
| 66 | Bone Morphogenetic Protein 2 | BMP2 | Glioma |
| 67 | lysyl oxidase | LOX | Lung |
| 68 | lectin galactoside-binding soluble, 4 | LGALS4 | Hepatocellular |
| 69 | Human Leucine-rich repeat and IQ domain-containing protein 4 | LRRIQ4 | Hepatocellular |
| 70 | Seven in absentia homolog 2 | SIAH2 | Hepatocellular |
| 71 | Glucosaminyl (N-Acetyl) Transferase 1 | GCNT1 | Hepatocellular |
| 72 | CACNAG1 | CACNAG1 | Colorectal |
| 73 | C7orf24 | C7orf24 | General |
| 74 | Inhibin pro-alphaC | Inhibin pro-alphaC | General |
| 75 | death receptor 3 | DR3 | Lung |
| 76 | Membrane-spanning 4 domain subfamily A from the multigene family of proteins involved in signal transduction of which CD20 is one member | MS4A | Lung |
| 77 | disulfide-isomerase | disulfide-isomerase | Hepatocellular |
| 78 | centromere protein F | centromere protein F | Hepatocellular |
| 79 | WD repeat domain 1 | WDR1 | Thyroid |
| 80 | osteoprotegerin | OPG | Ovarian |
| 81 | nicotinamide N-methyltransferase | NNMT | Renal |
| 82 | colon cancer specific antigen-2 | CCSA-2 | Colorectal |
| 83 | galectin-3 | GAL3 | Breast |
| 84 | PAK2 | PAK2; PAKgamma; PAK65 | Breast |
| 85 | Prohibitin-2 | PHB2 | Breast |
| 86 | Receptor for Activated Protein Kinase C | RACK1 | Breast |
| 87 | RUVBL1 | RUVBL1 | Breast |
| 88 | C9orf50-M | C9orf50-M | Colorectal |
| 89 | CLEC4D | CLEC4D | Colorectal |
| 90 | LMNB1 | LMNB1 | Colorectal |
| 91 | PRRG4 | PRRG4 | Colorectal |
| 92 | VNN1 | VNN1 | Colorectal |
| 93 | Heat shock protein 90 alpha | HSP90alpha | Lung |
| 94 | leucine-rich repeats and immunoglobulin-like domains 3 | LRIG3 | Lung |
| 95 | pleiotrophin | pleiotrophin | Lung |
| 96 | protein kinase C iota type | PRKCI | Lung |
| 97 | Repulsive Guidance Molecule C | RGM-C | Lung |
| 98 | Stem Cell Factor soluble Receptor | SCF-sR | Lung |
| 99 | YES | YES | Lung |
| 100 | Apo10 | Apo10 | Breast |
| 101 | TKTL1 | TKTL1 | Breast |
| 102 | Filamin B, beta | FLNB | Prostate |
| 103 | Dermokine-beta | DK-beta | Colorectal |
| 104 | Peripheral blood microvesicles | Peripheral blood MV | Hepatocellular |
| 105 | seprase | seprase | Colorectal |
| 106 | histidine-rich glycoprotein | HRG | Ovarian |
| 107 | SSBP2 | SSBP2 | Pancreatic |
| 108 | CA5B | CA5B | Pancreatic |
| 109 | F5 | F5 | Pancreatic |
| 110 | TBC1D8 | TBC1D8 | Pancreatic |
| 111 | ARG1 | ARG1 | Pancreatic |
| 112 | ADAM metallopeptidase with thrombospondin type 1 motif, 20 | ADAMTS20 | Pancreatic |
| 113 | death-associated protein kinase 1 | DAPK | Oesophageal |
| 114 | Human-Cervical-Cancer-Oncogene | HCCR | Hepatocellular |
| 115 | L-plastin | LPC1 | Renal |
| 116 | HMGB1 | HMGB1 | Mesothelioma |
| 117 | Tumor pyruvate kinase M2 | tumor M2-PK | Cervical, General |
| 118 | Heat shock protein 27 | HSP27 | Glioma, Pancreatic |
| 119 | Carbohydrate antigen 50 | CA50 | Lung |
| 120 | A1AG1 | A1AG1 | Hepatocellular |
| 121 | AACT | AACT | Hepatocellular |
| 122 | Ceruloplasmin | CERU | Hepatocellular |
| 123 | spondin-2 | spondin-2 | Ovarian, Prostate |
| 124 | transthyretin | TTR; TT | Endometrial, Gastric, Lung, Lymphoma, Ovarian |
| 125 | cytokeratin fragment 21.1 | cytokeratin fragment 21.1 | Lung |
| 126 | serum amyloid A | SAA | Colorectal, Lung, Ovarian, Pancreatic |
| 127 | Nidogen-2 | Nidogen-2 | Hepatocellular, Ovarian |
| 128 | carbohydrate antigen 211 | CA211 | Lung |
| 129 | c-terminal peptide crosslinks | CTX | Breast |
| 130 | osteocalcin | OC | Bone, Breast |
| 131 | apolipoprotein C-I | apolipoprotein C-I | Breast, Gastric, Prostate |
| 132 | lipocalin 2 | lipocalin 2 | Colorectal |
| 133 | highly sensitive fucosylated fraction of alpha-fetoprotein | hs-AFP-L3 | Hepatocellular |
| 134 | proprotein convertase subtilisin/kexin 6 | Pcsk6 | Prostate |
| 135 | PCSA | PCSA | Prostate |
| 136 | alpha-1-beta glycoprotein | A1BG | Pancreatic |
| 137 | serum amyloid p | SAP | Hepatocellular, Pancreatic |
| 138 | Y-box binding protein 1 | YB-1 | Hepatocellular |
| 139 | Niemann-Pick Type C2 | NPC2 | Hepatocellular |
| 140 | Cancer-testis antigen CT16 | CT16 | Melanoma |
| 141 | beta2-microglobulin | beta2M; beta2-MG; B2m | Hepatocellular, Myeloma, Oral, Prostate |
| 142 | thymidine kinase | TK1 | Leukemia |
| 143 | alpha1-antitrypsin | A1AT | Breast, Hepatocellular, Ovarian |
| 144 | chitinase 3-like 1 (cartilage glycoprotein-39) | YKL-40 | Ovarian |
| 145 | prostasin | prostasin | Ovarian |
| 146 | endoplasmic reticulum protein-29 | ERP29 | Lung |
| 147 | human carboxylesterase 1 | hCE1 | Hepatocellular |
| 148 | hyaluronic acid binding proteins | HABP | General |
