# Supplementary material for: Building the Evidence Base of Blood-Based Biomarkers for Early Detection of Cancer: A Rapid Systematic Mapping Review
Source: eBioMedicine. 2016 Jul 6;10:164–73. doi: 10.1016/j.ebiom.2016.07.004 (PMC5006664; doi:10.1016/j.ebiom.2016.07.004)
Supplement: Supplementary Table 11 — Nuclear proteins. [file mmc11.docx]

**Supplementary Table 11: Nuclear Proteins**

| No | Biomarker | Acronym | Cancer |
| --- | --- | --- | --- |
| 1 | p16 | p16 | Gastric, Hepatocellular, Oesophageal |
| 2 | k-ras | k-ras | Colorectal |
| 3 | isocitrate dehydrogenase 1 | IDH1 | Lung |
| 4 | p53 messenger RNA | p53 mRNA | Lung |
| 5 | Epstein-Barr virus nuclear antigen 1 | EBNA-1 | Nasopharyngeal carcinoma |
| 6 | p15 | p15 | Hepatocellular |
| 7 | nuclear receptor subfamily 2, group E, member 3 | NR2E3 | Pancreatic |
| 8 | SOX11 | SOX11 | Lymphoma |
| 9 | microphthalmia melanoma | MITF-M | Melanoma |
| 10 | E2F6 | E2F6 | Lung |
| 11 | heterogeneous nuclear ribonucleoproteins F | hnRNPF | Breast |
| 12 | Transciption initiation factor-like | TFIID-like | Hepatocellular |
| 13 | Variant Ciz1 | Ciz1 | Lung |
