# Supplementary material for: Building the Evidence Base of Blood-Based Biomarkers for Early Detection of Cancer: A Rapid Systematic Mapping Review
Source: eBioMedicine. 2016 Jul 6;10:164–73. doi: 10.1016/j.ebiom.2016.07.004 (PMC5006664; doi:10.1016/j.ebiom.2016.07.004)
Supplement: Supplementary Table 12 — Microbial proteins. [file mmc12.docx]

**Supplementary Table 12: Microbial Proteins**

| No. | **Biomarker** | **Acronym** | **Cancer** |
| --- | --- | --- | --- |
| 1 | Epstein-Barr Virus latent membrane protein 2A | EBV LMP2A | General |
| 2 | Epstein-Barr Virus DNA | EBV DNA; EBV-DNA | Lymphoma, Nasopharyngeal carcinoma |
| 3 | Epstein-Barr virus early antigen | EBV EA | Nasopharyngeal carcinoma |
| 4 | Epstein-Barr nuclear antigen-1 IgA | EBNA-1 IgA | Nasopharyngeal carcinoma |
| 5 | Epstein-Barr virus viral capsid antigen IgA | EBV VCA IgA | Nasopharyngeal carcinoma |
| 6 | Epstein-Barr virus-induced gene 3 | EBI3 | Lung |
| 7 | Antibodies HPV16 E1 | Abs HPV16 E1 | Oral |
| 8 | Antibodies HPV16 E2 | Abs HPV16 E2 | Oral |
| 9 | Antibodies HPV16 E4 | Abs HPV16 E4 | Oral |
| 10 | Antibodies HPV16 E6 | Abs HPV16 E6 | Oral |
| 11 | Antibodies HPV16 E7 | Abs HPV16 E7 | Oral |
| 12 | Antibodies HPV16 L1 | Abs HPV16 L1 | Oral |
| 13 | H Pylori Outer membrane protein antibodies | OMP | Gastric |
| 14 | Helicobacter pylori-0305 | HP0305 | Gastric |
| 15 | H Pylori vacuolating toxin | VacA | Gastric |
