# Supplementary material for: Building the Evidence Base of Blood-Based Biomarkers for Early Detection of Cancer: A Rapid Systematic Mapping Review
Source: eBioMedicine. 2016 Jul 6;10:164–73. doi: 10.1016/j.ebiom.2016.07.004 (PMC5006664; doi:10.1016/j.ebiom.2016.07.004)
Supplement: Supplementary Table 13 — Volatile organic compounds. [file mmc13.docx]

**Supplementary Table 13: Volatile Organic Compounds**

| **No** | **Biomarker** | **Acronym** | **Cancer** |
| --- | --- | --- | --- |
| 1 | phenyl methylcarbamate | phenyl methylcarbamate | Colorectal |
| 2 | ethylhexanol | ethylhexanol | Colorectal |
| 3 | 6-t-butyl-2,2,9,9-tetramethyl-3,5- decadien-7-yne | 6-t-butyl-2,2,9,9-tetramethyl-3,5- decadien-7-yne | Colorectal |
